# Cytolytic CD8^+^ T cells infiltrate germinal centers to limit ongoing HIV replication in spontaneous controller lymph nodes

**DOI:** 10.1126/sciimmunol.ade5872

**Published:** 2023-05-19

**Authors:** David R. Collins, Julia Hitschfel, Jonathan M. Urbach, Geetha H. Mylvaganam, Ngoc L. Ly, Umar Arshad, Zachary J. Racenet, Adrienne G. Yanez, Thomas J. Diefenbach, Bruce D. Walker

**Affiliations:** 1 Ragon Institute of MGH, MIT and Harvard, Cambridge, MA, USA; 2 Howard Hughes Medical Institute, Chevy Chase, MD, USA; 3 Institute of Clinical and Molecular Virology, Friedrich-Alexander Universität Erlangen-Nürnberg, Erlangen, Germany; 4 Institute for Medical Engineering and Sciences and Department of Biology, Massachusetts Institute of Technology, Cambridge, MA, USA

## Abstract

Follicular CD8^+^ T cells (fCD8) mediate surveillance in lymph node (LN) germinal centers against lymphotropic infections and cancers, but precise mechanisms by which these cells mediate immune control remain incompletely resolved. To address this, we investigated functionality, clonotypic compartmentalization, spatial localization, phenotypic characteristics, and transcriptional profiles of LN-resident virus-specific CD8^+^ T cells in persons who control HIV without medications. Antigen-induced proliferative and cytolytic potential consistently distinguished spontaneous controllers from noncontrollers. T cell receptor analysis revealed complete clonotypic overlap between peripheral and LN-resident HIV-specific CD8^+^ T cells. Transcriptional analysis of LN CD8^+^ T cells revealed gene signatures of inflammatory chemotaxis and antigen-induced effector function. In HIV controllers, the cytotoxic effectors perforin and granzyme B were elevated among virus-specific CXCR5^+^ fCD8s proximate to foci of HIV RNA within germinal centers. These results provide evidence consistent with cytolytic control of lymphotropic infection supported by inflammatory recruitment, antigen-specific proliferation, and cytotoxicity of fCD8s.

## ONE-SENTENCE SUMMARY

Antigen-driven follicular CD8^+^ T cell cytotoxicity contributes to immune control of HIV replication within germinal centers.

## INTRODUCTION

Lymph nodes (LN) are highly structured secondary lymphoid organs that support the induction of adaptive immune responses through tightly regulated cell-cell interactions (1, 2). CXCR5^+^ B cells and follicular helper CD4^+^ T cells home toward CXCL13 produced by follicular dendritic cells and interact within germinal centers (GC) to facilitate antibody maturation, whereas naive T cells traffic to the LN paracortex via CCR7-mediated chemotaxis toward CCL19 and CCL21 produced by fibroblastic reticular cells (3). Naïve T cells become primed by antigen-presenting cells in LNs and recirculate through lymph via sphingosine-1 phosphate-mediated egress (4), which is inhibited by CD69 on activated and LN-resident cells (5, 6).

In addition to their functions in generating immune responses, LNs are also targeted by lymphotropic viral infections, neoplasms and metastases, necessitating a careful balance between immune privilege and surveillance. Whereas pathogens in nonlymphoid tissues are typically contained or cleared by tissue-resident and circulating cytotoxic CD8^+^ T cells (7, 8), follicular GCs within lymphoid tissues represent a semi-privileged niche with limited immune surveillance (9). Nonetheless, accumulation of follicular CD8^+^ T cells (fCD8) has been associated with immune control of pathogenic viral infections and cancers, including LCMV (10–12), HIV (11– 16), SIV (17–21), EBV infectious mononucleosis (12, 22–24), B cell lymphoma (25–27) and HBV-related carcinoma (28), as well as lung (29), pancreatic (30) and colorectal cancers (31, 32).

Despite these associations of fCD8s with immune control, effector mechanisms employed by these cells are not fully defined. Unlike most circulating CD8^+^ T cells, fCD8s express CXCR5 and lack CCR7, thus enabling access to GCs (33). fCD8s express low levels of the cytotoxic effector molecules perforin and granzyme B in healthy tissue and can exert both supportive and suppressive effects on immune responses (33–35). The precise mechanisms by which fCD8s contribute to long-term immunity within infected LNs remain incompletely resolved, including determinants of their compartmentalization, recruitment, and functional regulation.

Spontaneous control of HIV infection in the absence of antiviral therapy, in which virus is not eliminated but actively held in check, provides an opportunity to more precisely determine the antiviral properties of fCD8s. In spontaneous HIV controllers, CD8^+^ T cell-mediated immunity has been repeatedly associated with containment of viremia in studies of peripheral blood (PB) (36), but HIV replication primarily persists within LN follicular helper CD4^+^ T (T_FH_) cells in these individuals (37, 38), as it does in uncontrolled chronic infection (39–44) and during antiretroviral therapy (ART) (45–49). Limited GC access of cytotoxic CD8^+^ T cells is thought to contribute to this persistent viral replication (13, 19, 20, 50–56). The extent to which cytolytic activity of fCD8s mediates viral containment remains controversial, as expression of perforin and granzymes in studies of LN-resident CD8^+^ T cells has been variable (10–14, 17–19, 53–69). This variability suggests that fCD8 cytotoxicity is modulated by contextual factors that remain to be elucidated, such as pathogen burden, disease stage and/or immune dysfunction.

Here we examined lymphoid CD8^+^ T cells from persons with durable spontaneous control of HIV replication to elucidate mechanisms by which these cells sustain immune control of active HIV replication within LNs. We investigated their *in situ*, *ex vivo* and antigen-induced functionality in paired LN and PB obtained from long-term spontaneous controllers with a range of ongoing LN HIV replication. In addition, we investigated spatial localization, clonotypic compartmentalization, and transcriptional differences associated with localization of HIV-specific CD8^+^ T cells within PB and LN, providing insights into regulation of migration and cytotoxicity in LNs during long-term immune control of a chronic lymphotropic viral infection.

## RESULTS

### HIV-specific CD8^+^ T cells in controller LNs proliferate and acquire cytotoxicity upon antigen stimulation

We obtained contemporaneous PB and excisional inguinal LN specimens from 43 study participants, including 19 spontaneous HIV controllers, 17 ART-suppressed HIV noncontrollers, and 7 HIV-negative individuals (table 1). Among controllers, 8 had undetectable plasma viremia (elite control) and 11 had low-level detectable viremia (viremic control) with a median of 72 HIV RNA copies/ml plasma at the time of sampling, whereas noncontrollers had a median peak viral load of 300,000 copies/mL prior to pharmacologic suppression for a median of 12 years. Irrespective of plasma viral load, all spontaneous controllers maintained suppression of viremia to below 2,000 RNA copies/ml for a median of 23 years (range 8–33 years) prior to sampling.

To facilitate downstream analyses, we first mapped HIV antigen-specific CD8^+^ T responses by screening peripheral blood mononuclear cells (PBMCs) for interferon-gamma (IFN-γ) secretion by enzyme-linked immunospot (ELISPOT) assay following stimulation with arrays of HLA-optimal consensus clade B HIV peptides (listed in table S1). We detected a median of 3 HIV epitope-specific responses per subject, then confirmed immunodominant responses (listed in table S2) by flow cytometric assessment of peptide-HLA (pHLA) tetramer binding (fig. S1). In agreementwith prior studies (61, 70–72), we observed similar frequencies of HIV-specific CD8^+^ T cells among PB and LN.

**Fig. 1.**
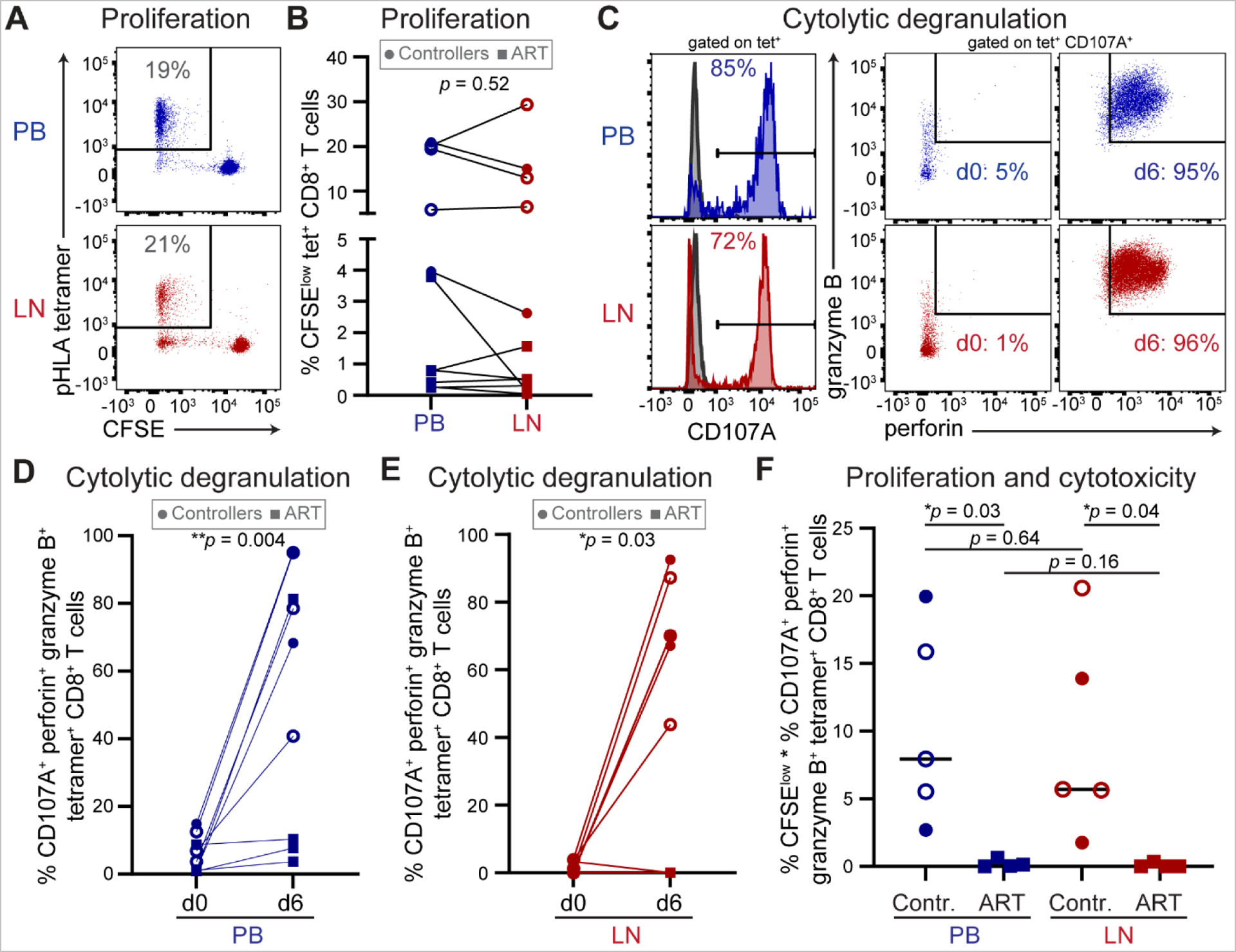
HIV-specific CD8^+^ T cells isolated from spontaneous controller LNs proliferate and develop cytolytic function upon antigenic stimulation. **(A)** Representative proliferation as measured by CFSE dilution in CD8^+^ T cells from EC PB or LN upon 6-day stimulation with HLA-optimal HIV peptide followed by staining with cognate pHLA tetramer. **(B)** Summary of PB and LN HIV-specific CD8^+^ T cell proliferation (*n* = 11). Each data point represents the average of triplicate values. Wilcoxon matched-pairs signed rank test was used to calculate *p* value. **(C)** Representative HIV antigen-specific degranulation as measured by surface CD107A upregulation relative to unstimulated controls (gray histograms, left panels) and co-expression of intracellular perforin and granzyme B upon 4-hour stimulation of CD8^+^ T cells from EC PB and LN with cognate pHLA tetramer before (d0, middle panels) and after (d6, right panels) peptide expansion, gated on pHLA tetramer^+^ CD107A^+^ cells. **(D-E)** Summary of cytotoxicity as measured in C among PB (D) and LN (E) HIV-specific pHLA tetramer^+^ CD8^+^ T cells before (d0) and after (d6) peptide-specific proliferation (*n* = 9). Wilcoxon matched-pairs signed rank tests were used to calculate *p* values. **(F)** Summary of combined ability of HIV-specific CD8^+^ T cells to proliferate and acquire cytotoxicity as measured by the product of proliferation (as measured in A-B) and d6 cytotoxicity (as measured in C-E) among controllers (*n* = 5, circles) and ART-suppressed noncontrollers (*n* = 4, squares). Lines represent medians. Paired (PB vs LN) and unpaired (controllers vs ART) *t* tests were used to calculate *p* values. Gray boxes contain symbol keys: circles represent controller, squares ART; open or closed circles denote controllers with undetectable or detectable plasma HIV RNA, respectively.

Proliferative and cytotoxic potential upon antigen stimulation is a major functional correlate of durable spontaneous control of viremia among HIV-specific CD8^+^ T cells in PB (73–79). To examine whether CD8^+^ T cells isolated from lymphoid tissues of HIV controllers have similar functional attributes, we measured the capacities of LN and PB HIV-specific CD8^+^ T cells to proliferate, express the cytolytic effector molecules perforin and granzyme B, and degranulate upon antigen re-exposure. After 6-day stimulation of carboxyfluorescein succinimidyl ester (CFSE)-labeled cells with immunodominant HLA-optimal peptide, specificity was reconfirmed using matched pHLA tetramer staining (fig. 1A). PB and LN HIV-specific CD8^+^ T cells exhibited similar proliferative capacities (fig. 1B).

To assess cytotoxic potential, we measured degranulation (via surface CD107A upregulation) and co-expression of perforin and granzyme B among HIV-specific CD8^+^ T cells stimulated for 4 hours with pHLA tetramer before and after 6-day peptide-specific expansion (fig. 1C). Compared to cytolytic degranulation before expansion, which was low among HIV-specific CD8^+^ T cells in both PB and LN, we observed large increases in cytotoxicity upon HIV antigen-induced proliferation in both PB (fig. 1D) and LN (fig. 1E). The combined ability of HIV-specific CD8^+^ T cells to proliferate and upregulate cytotoxic effector molecules was higher among spontaneous controllers relative to ART-suppressed noncontrollers in both PB and LN (fig. 1F). These data indicate that circulating and lymphoid tissue-derived HIV-specific CD8^+^ T cells from HIV controllers share robust potential for expansion and cytotoxicity compared to ART-treated noncontrollers.

### LN and circulating HIV-specific CD8^+^ T cell populations share T cell receptor clonotypic composition

**Fig. 2.**
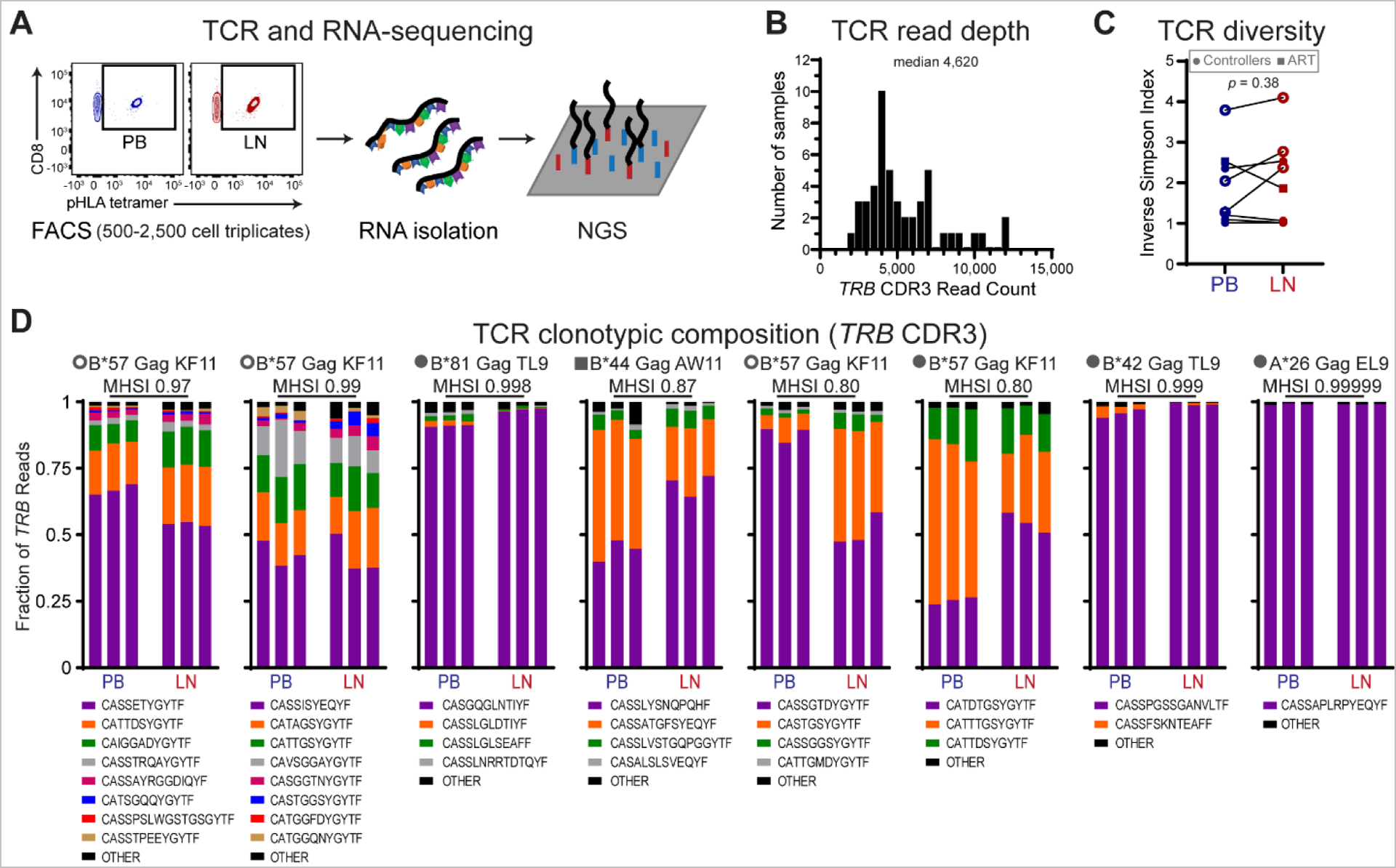
HIV-specific CD8^+^ T cells in PB and LN share clonotypic composition. **(A)** Schematic overview of fluorescence-activated cell sorting (FACS) isolation of triplicate populations of 500–2,500 HIV pHLA tetramer^+^ CD8^+^ T cells from matched PB and LN specimens, followed by RNA isolation and next-generation sequencing (NGS). **(B)** Summary metrics for T cell receptor (TCR) beta gene (*TRBC*) CDR3 read depth per sample. **(C)** TCR diversity as measured by inverse Simpson diversity index in matched PB and LN from 8 individuals. Gray box contains symbol key: circles represent controllers (*n* = 7) and square ART (*n* = 1); open or closed circles denote controllers with undetectable or detectable plasma HIV RNA, respectively. Paired t-test was used to calculate *p* value. **(D)** Clonotypic composition of HIV-specific pHLA tetramer^+^ CD8^+^ T cells as measured by *TRBC* CDR3 sequences among triplicate matched PB and LN samples from 8 individuals as in C. MHSI, Morisita-Horn Similarity Index.

Given the similarity between PB and LN T cells in frequency and response to antigen, we next explored the extent of HIV-specific CD8^+^ T cell compartmentalization in LN compared to PB. We sorted triplicate populations of 500–2,500 unstimulated HIV pHLA tetramer^+^ CD8^+^ T cells from contemporaneous, matched LN and PB and performed RNA sequencing using paired-end, 75 base length reads to allow efficient mapping of T cell receptor (TCR) sequences (fig. 2A). We obtained a median of 4,620 TCR beta (*TRB*) complementarity-determining region 3 (CDR3) sequences per replicate sample (fig. 2B), allowing for robust clonotypic characterization of HIV-specific CD8^+^ T cells. TCR diversity was comparable between LN and PB HIV-specific CD8^+^ T cells (fig. 2C). Clonotypic identity was shared between circulating and tissue-derived HIV-specific CD8^+^ T cells, with no unique clonotypes detected in either PB or LN from any of the eight individuals sampled (fig. 2D). Moreover, clonotypes were present at similar frequencies in LN and PB, with highly similar oligoclonal compositions quantified by Morisita-Horn similarity index (MHSI) (fig. 2D). These data indicate a lack of discrete clonotypic segregation between HIV-specific populations of CD8^+^ T cells in LN and PB.

### Chemotactic and effector transcriptional signatures are elevated in LN HIV-specific CD8^+^ T cells

**Fig. 3.**
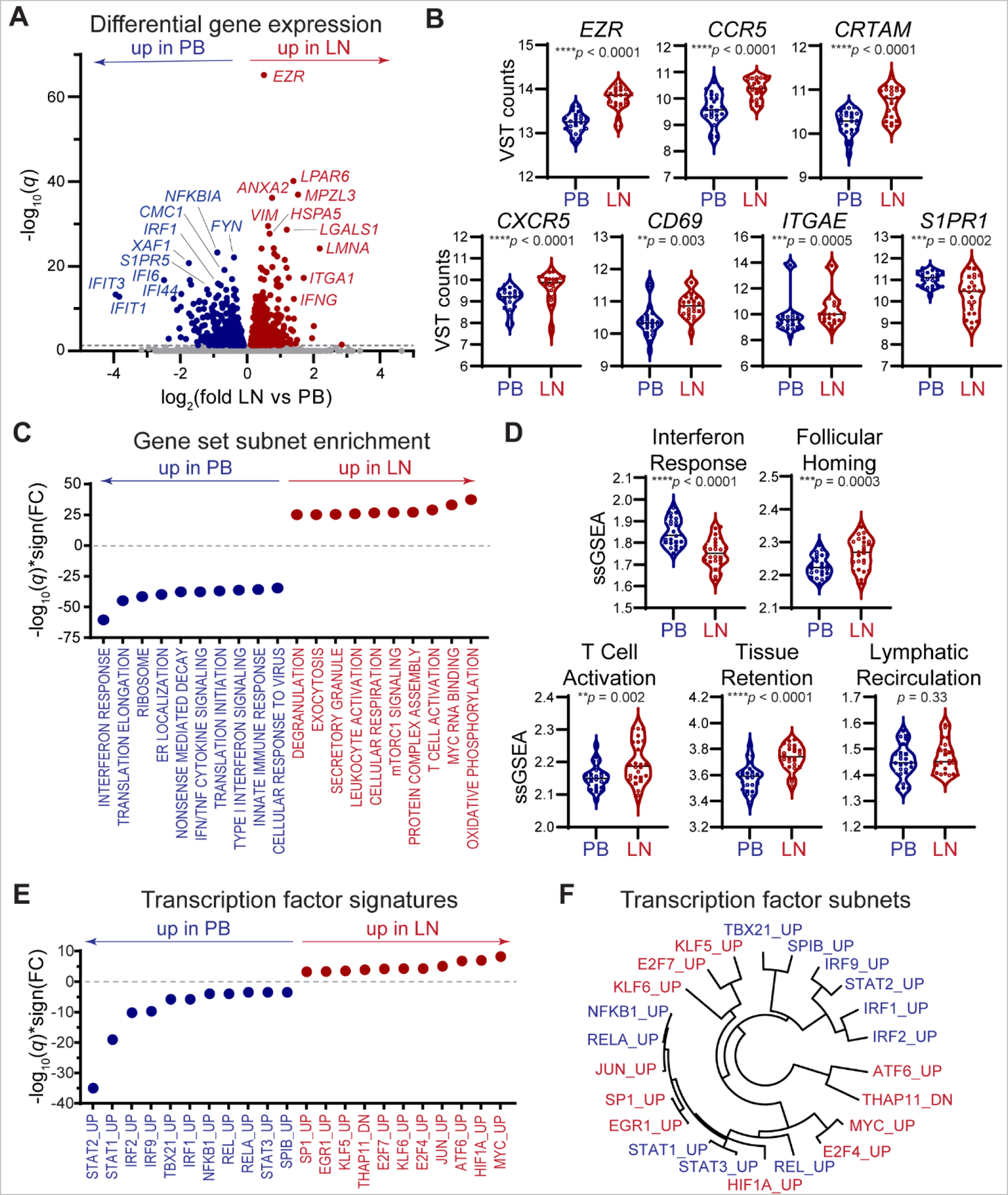
Transcriptional profiling of HIV-specific CD8^+^ T cells reveals chemotactic and effector signatures in LN. **(A)** Volcano plot summarizing differential gene expression between PB and LN HIV-specific CD8^+^ T cells isolated from 8 participants as in Fig. 2A. Dashed line represents significance cutoff (*q* = 0.05). **(B)** Violin plots of VST-normalized counts for select differentially expressed genes in PB and LN. Each data point represents one replicate sample, circles represent controllers (*n* = 7) and square ART (*n* = 1); open or closed circles denote controllers with undetectable or detectable plasma HIV RNA, respectively. Paired t-tests were used to calculate *p* values. **(C)** Summary of top ten most significantly differentially expressed gene set subnets enriched in LN and in PB. **(D)** Single-sample gene set enrichment analyses (ssGSEA) for genes associated with interferon responses, chemotactic CD8^+^ LN follicular homing, virus-specific CD8^+^ T cell activation, CD8^+^ T cell lymphoid tissue retention and lymphoid-emigrant CD8^+^ T cell tissue recirculation. Each data point represents one sample, circles represent controllers (*n* = 7) and square ART (*n* = 1); open or closed circles denote controllers with undetectable or detectable plasma HIV RNA, respectively. Paired t-tests were used to calculate *p* values. **(E)** Summary of inferred transcription factor activity significantly enriched in LN and in PB. **(F)** Hierarchical dendrogram summarizing relationships between inferred transcriptional regulators significantly enriched in LN (red) and PB (blue).

We performed deep transcriptional profiling of unstimulated HIV-specific CD8^+^ T cells in LN and PB using the RNA-seq approach described above in the same eight participants. We analyzed a median of 14,034 unique genes per replicate sample (range 11,337–14,849) for differential expression, of which 727 genes were upregulated in LN and 620 in PB (fig. 3A, table S3). The most upregulated gene among LN HIV-specific CD8^+^ T cells, *EZR*, has been previously shown to promote T cell homing to lymphoid organs (80). Genes involved in inflammatory chemotaxis, follicular homing, T cell activation and tissue retention, including elevated *CCR5*, *CXCR5*, *CRTAM, CD69*, *ITGAE* (CD103), and decreased *S1PR1*, were also differentially expressed in LN relative to PB (fig. 3B). We applied gene set network analysis (79) to more comprehensively delineate biological processes differentiating HIV-specific CD8^+^ T cells in PB and LN (fig. S2). Transcriptional programs associated with T cell activation, proliferation and cytotoxicity, including oxidative phosphorylation (81), Myc RNA binding (82), mTORC1 signaling (83) and cytolytic degranulation were elevated in LN, whereas genes involved in innate immune activation via interferon (IFN) and tumor necrosis factor (TNF) signaling were enriched in PB (fig. 3C, table S4). Single-sample gene set enrichment analyses (ssGSEA) further demonstrated that LN localization of HIV-specific CD8^+^ T cells was associated with a decreased interferon response and elevated signatures of follicular homing (12), virus-specific activation (84) and tissue retention (85), but not recirculation through lymphatic vessels (86) (fig. 3D).

We applied enrichment analysis using curated transcription factor-associated gene sets (87) to define a network of upstream transcriptional activators and repressors in PB and LN HIV-specific CD8^+^ T cells (fig. 3E–F). Circulating HIV-specific CD8^+^ T cells expressed genes regulated by STAT, IRF and NFKB family transcription factors, consistent with inflammatory cytokine signaling, and by TBX21/T-bet, which was previously shown to mediate expansion and differentiation of CD8^+^ T cells in response to inflammatory cytokines (88) and to induce CXCR3-mediated inflammatory chemotaxis (89). Conversely, LN-derived CD8^+^ T cells expressed genes regulated by Myc, HIF1A and JUN/AP-1 transcription factors, consistent with antigen-driven T cell activation within a hypoxic inflamed tissue microenvironment (82, 90) and by EGR1, which promotes antigen-driven CD8^+^ T cell proliferation in an AP-1-dependent manner (91). Together with clonotypic data shown in fig. 2, these results suggest the possibility that HIV-specific CD8^+^ T cell clones may migrate from PB to LN via inflammatory chemotaxis, become activated by TCR-pHLA antigen recognition, and express genes involved in cytolytic effector function and tissue retention, potentially replenishing and augmenting tissue resident cellular immunity within HIV-infected LNs.

### HIV-specific fCD8s express cytolytic effectors and LN retention receptors in spontaneous controllers

**Fig. 4.**
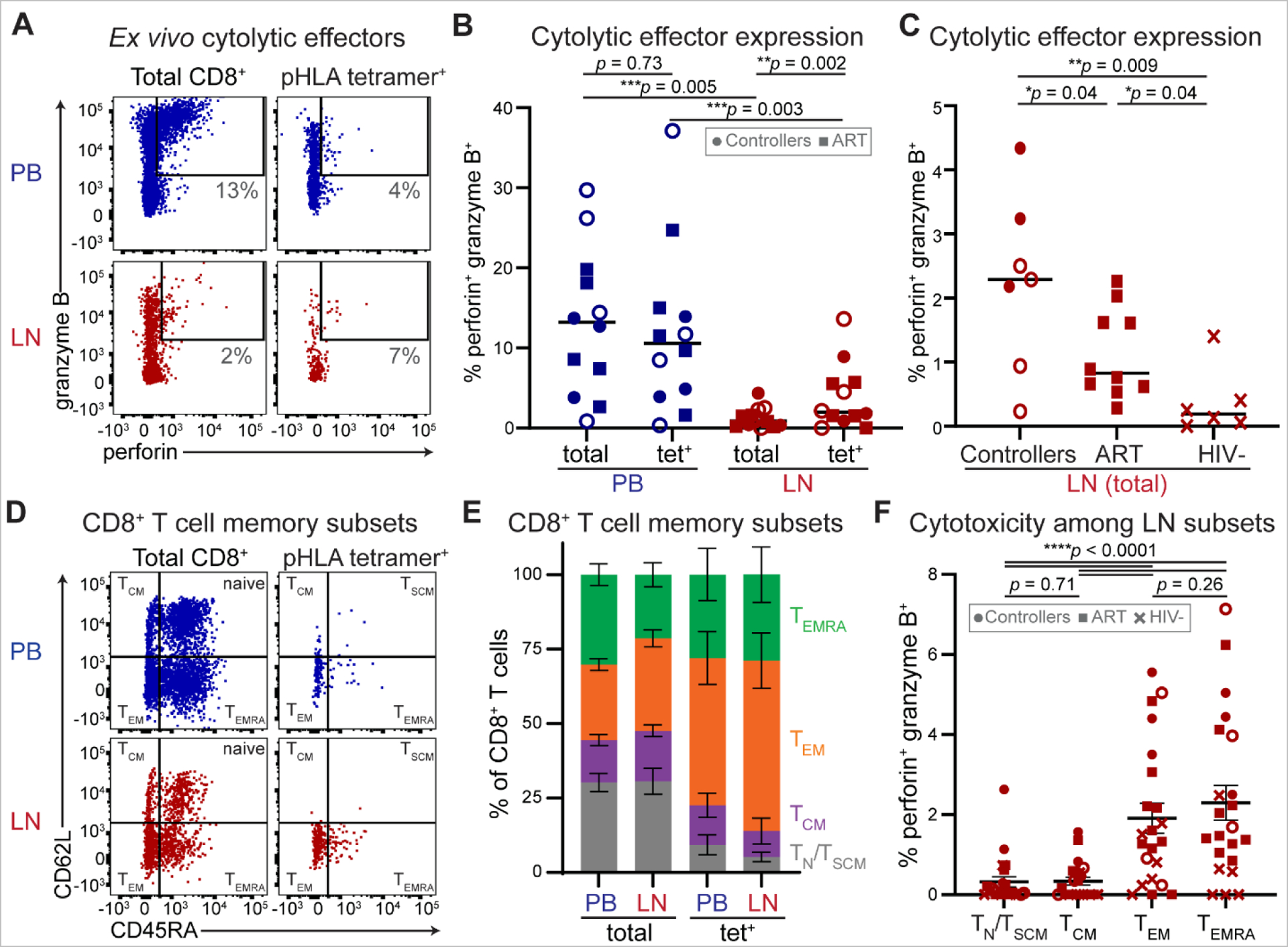
Cytolytic HIV-specific effector-memory CD8^+^ T cells are elevated in LN during HIV control. **(A)** Representative *ex vivo* intracellular perforin and granzyme B expression in total and pHLA tetramer^+^ CD8^+^ T cells from PB and LN of a controller. **(B)** Summary of *ex vivo* perforin and granzyme B expression among total and HIV-specific pHLA tetramer^+^ (tet^+^) CD8^+^ T cells in PB and LN in HIV^+^ individuals (*n* = 12). Lines represent medians. Wilcoxon matched-pairs signed rank tests were used to calculate *p* values. **(C)** Summary of perforin and granzyme B expression among total LN CD8^+^ T cells among HIV controllers (*n* = 7, circles), ART-suppressed noncontrollers (*n* = 10, squares) and HIV-negative individuals (*n* = 6; crosses). Lines represent medians. Unpaired *t* tests were used to calculate *p* values. **(D)** Representative surface CD45RA and CD62L memory subset marker expression on total or pHLA tetramer^+^ CD8^+^ T cells from PB and LN. **(E)** Summary of memory subset composition among total (*n* = 22) and HIV-specific pHLA tetramer^+^ (*n* = 8) CD8^+^ T cells in PB and LN. Stacked bars represent mean, error bars represent 95% confidence intervals. **(F)** Frequencies of dual perforin and granzyme B expression among CD8^+^ T cell memory subsets in LN (*n* = 22). Lines represent medians. Wilcoxon matched-pairs signed rank tests were used to calculate *p* values. Gray boxes contain symbol keys: circles represent controllers, squares ART and crosses HIV-; open or closed circles denote controllers with undetectable or detectable plasma HIV RNA, respectively.

To validate and further assess the roles of inflammatory chemotaxis, follicular homing, tissue retention and cytotoxicity in control of lymphoid HIV replication at single-cell resolution, we measured protein expression of key mediators of these processes among total and HIV-specific pHLA tetramer^+^ CD8^+^ T cells in LN and PB by flow cytometry. Consistent with prior reports (13, 14), *ex vivo* expression of the cytotoxic effector molecules granzyme B and perforin were lower among LN CD8^+^ T cells compared to PB (fig. 4A). Nonetheless, perforin and granzyme B were consistently detected within LN and were elevated among HIV-specific relative to total CD8^+^ T cells in LN (fig. 4B). In both compartments, perforin expression was limiting and was almost exclusively detected within granzyme B-expressing cells (fig. S3A**-**B). LN CD8^+^ T cells also expressed more perforin and granzyme B in HIV-infected relative to HIV-uninfected persons, with higher expression in HIV controllers than in ART-suppressed individuals (fig. 4C). Taken together with results shown in fig. 1, these data are consistent with cytolytic effector molecule expression induced by antigen-driven CD8^+^ T cell proliferation in HIV controller LNs.

To evaluate memory subset phenotypes of PB and LN cells, we stained for surface expression of CD45RA and CD62L (fig. 4D) or CCR7 (fig. S3C). HIV-specific CD8^+^ T cell populations were most enriched for an effector-memory phenotype in both LN and PB (fig. 4E). Effector-memory (T_EM_) and CD45RA^+^ terminally differentiated effector-memory (T_EMRA_) subsets expressed higher levels of perforin and granzyme B than central memory (T_CM_) or naïve/stem-like memory (T_NAIVE_*/*T_SCM_) CD8^+^ T cells in LN (fig. 4F). The marked similarity in CD8^+^ T cell memory phenotype in PB and LN further suggests minimal compartmentalization, possibly resulting from inflammatory recruitment of circulating effector-memory cells with cytolytic potential to infected LNs.

**Fig. 5.**
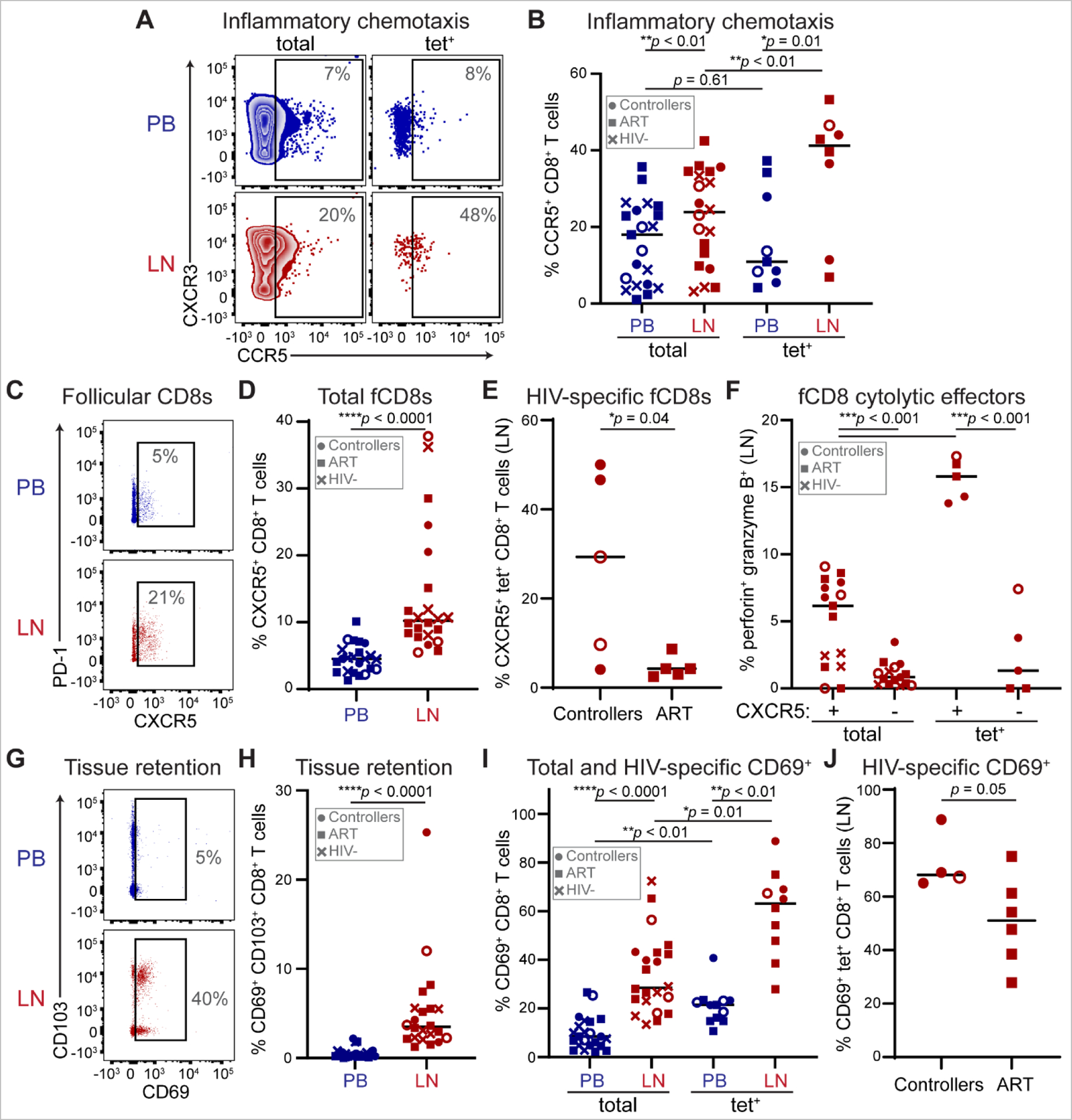
Cytolytic fCD8s are retained in spontaneous controller LNs. **(A)** Representative surface CCR5 and CXCR3 staining and gating on total and HIV-specific pHLA tetramer^+^ CCR5^+^ CD8^+^ T cells in PB and LN. **(B)** Summary of CCR5 expression among total (*n* = 21) and HIV-specific pHLA tetramer^+^ (*n* = 9) CD8^+^ T cells in PB and LN. Lines represent medians. Paired t-tests were used to calculate *p* values. **(C)** Representative surface CXCR5 and PD-1 staining and gating on CXCR5^+^ CD8^+^ T cells in PB and LN. **(D)** Summary of CXCR5 expression among CD8^+^ T cells from paired PB and LN (*n* = 22). Lines represent medians. Wilcoxon matched-pairs signed rank test was used to calculate *p* value. **(E)** Summary of CXCR5 expression among HIV-specific pHLA tetramer^+^ CD8^+^ T cells in spontaneous controllers (*n* = 5) and ART-suppressed non-controllers (*n* = 5). Lines represent medians. Unpaired t test was used to calculate *p* value. **(F)** Summary of perforin and granzyme B co-expression among CXCR5^+^ and CXCR5− subsets of total (*n* = 15) and HIV-specific pHLA tetramer^+^ (*n* = 5) LN CD8^+^ T cells. Lines represent medians. Paired t tests were used to calculate *p* values.**(G)** Representative surface CD69 and CD103 staining and gating on CD69^+^ CD8^+^ T cells in PB and LN. **(H)** Summary of CD69 and CD103 co-expression among CD8^+^ T cells from paired PB and LN (*n* = 22). Lines represent medians. Wilcoxon matched-pairs signed rank test was used to calculate *p* value. **(I)** Summary of CD69 expression among total (*n* = 22) and HIV-specific pHLA tetramer^+^ (*n* = 10) CD8^+^ T cells in PB and LN. Lines represent medians. Wilcoxon matched-pairs signed rank tests were used to calculate *p* values. **(J)** Summary of CD69 expression among LN HIV-specific pHLA tetramer^+^ CD8^+^ T cells in spontaneous controllers (*n* = 4) and ART-suppressed non-controllers (*n* = 6). Lines represent medians. Unpaired t test was used to calculate *p* value. Gray boxes contain symbol keys: circles represent controllers, squares ART and crosses HIV-; open or closed circles denote controllers with undetectable or detectable plasma HIV RNA, respectively.

We next evaluated surface protein expression of the inflammatory chemokine receptors CCR5 and CXCR3. CCR5^+^ cells generally co-expressed CXCR3 (fig. 5A), were elevated among LN CD8^+^ T cells, and were further elevated among HIV-specific CD8^+^ T cells in LN (fig. 5B). In contrast, the fractalkine receptor CX3CR1 was elevated among PB CD8^+^ T cells (fig. S3D), consistent with the relative exclusion of CX3CR1^hi^ T cells from LNs (92) and suggesting minimal contribution of blood vessel-derived cells to our observed LN CD8^+^ T cell phenotypes. These results suggest a role for CCR5 and CXCR3 in migration from PB to LN, consistent with transcriptional analyses in fig. 3.

The follicular homing receptor CXCR5 was expressed at a higher frequency in LN compared to PB (fig. 5C–D), as expected. Consistent with prior reports (13–18), CXCR5^+^ fCD8s were more prevalent among LN HIV-specific CD8^+^ T cells from spontaneous controllers than noncontrollers (fig. 5E), further implicating their importance in sustaining long-term control of follicular viral replication. Moreover, we observed higher perforin and granzyme B expression among CXCR5^+^ fCD8s compared to CXCR5^−^ CD8^+^ T cells in LN, with further elevated cytolytic effector molecule expression among HIV-specific fCD8s relative to total LN CD8^+^ T cells (fig. 5F), consistent with antiviral cytolytic potential of HIV-specific fCD8s.

We additionally measured expression of activation and tissue retention markers. CD69 and CD103, which are induced by T cell activation and mediate tissue retention, were higher among CD8^+^ T cells from LN relative to PB (fig. 5G–H). CD69 was further elevated among HIV-specific pHLA tetramer^+^ relative to total CD8^+^ T cells in both compartments (fig. 5I). CD69 was elevated among CXCR5^+^ fCD8s (fig. S3E), which were present at a higher frequency of HIV-specific CD8^+^ T cells in controllers (fig. 5E, J). These results are consistent with the retention of activated fCD8s during spontaneous control of LN infection.

Taken together, these data demonstrate that HIV-specific CD8^+^ T cells express molecules associated with inflammatory chemotaxis, follicular homing, activation, cytotoxic effector function and tissue retention in spontaneous controller LNs, enabling cytolytic control of viral replication within follicular GCs.

### fCD8s express cytotoxic effector molecules proximate to HIV replication in spontaneous controller GCs

**Fig. 6.**
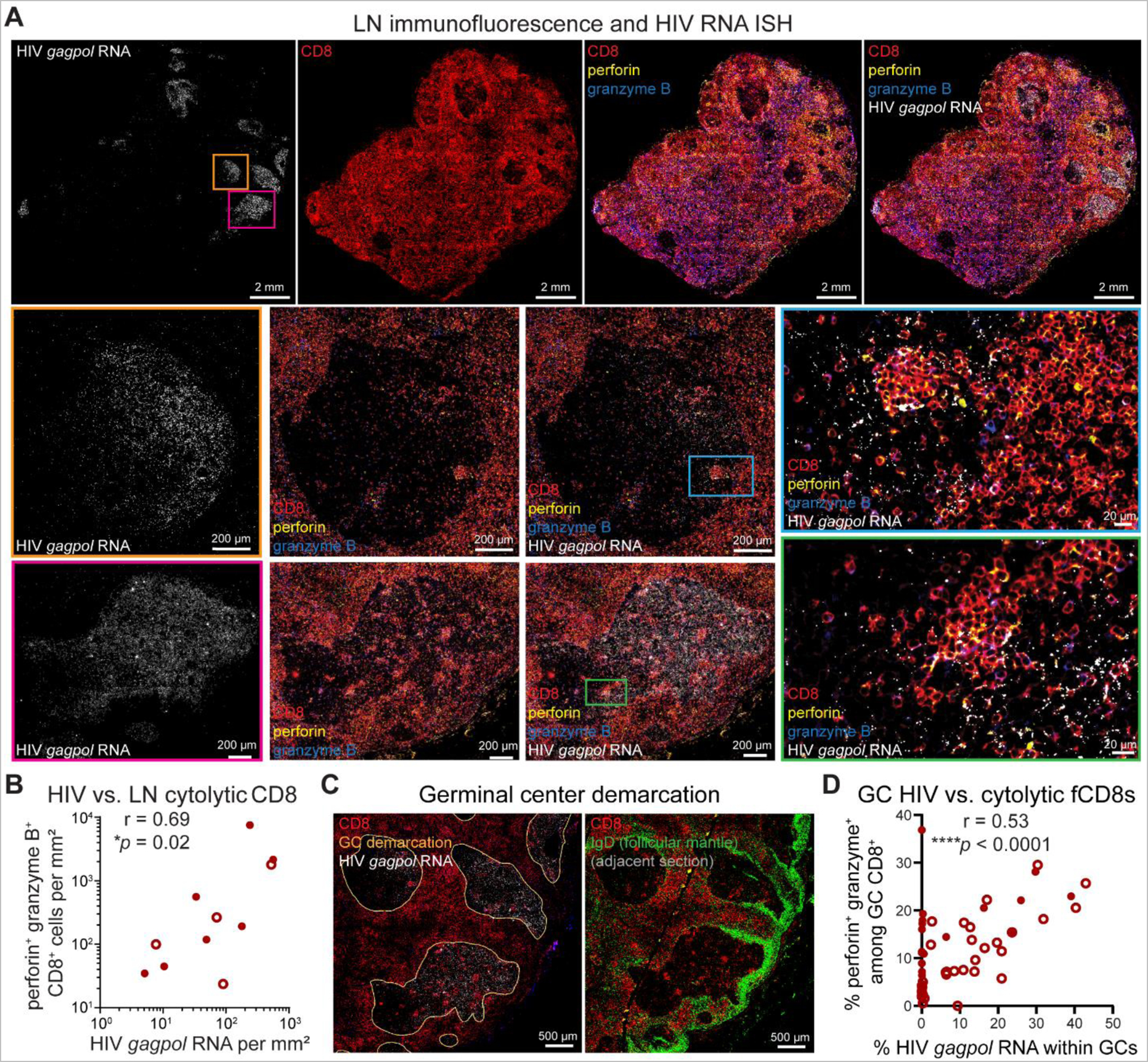
Cytolytic CD8^+^ T cells are present in controller LNs during HIV replication. **(A)** Representative immunofluorescence (IF) and RNAscope micrographs of spontaneous controller LN stained for HIV *gagpol* RNA (white), CD8 (red), perforin (yellow) and granzyme B (blue). Cellular interactions within follicular GCs are highlighted by enlarged insets. **(B)** Spearman correlation between density of perforin/granzyme B co-expressing CD8^+^ cells and density of cells expressing HIV *gagpol* RNA across entire LN tissue cross-sections. Each data point represents one HIV controller (*n* = 11) ; open or closed circles denote controllers with undetectable or detectable plasma HIV RNA, respectively. **(C)** Follicular GC regions of interest (ROI) demarcated by orange lines based upon morphology, relative CD8 exclusion (red), and IgD staining (green) of adjacent sections. **(D)** Spearman correlation between frequencies of perforin/granzyme B co-expressing CD8^+^ cells and of HIV *gagpol* RNA expressing cells within follicular GCs. Each data point represents one follicular GC (*n* = 52) from 6 total HIV controller LNs; open or closed circles denote GCs from controllers with undetectable or detectable plasma HIV RNA, respectively.

We next assessed spatial relationships between active HIV replication in LN and cytolytic effector molecule expression among CD8^+^ T cells. We hypothesized that, similar to PB where recent antigen exposure is a key determinant of the *ex vivo* effector function of CD8^+^ T cells from HIV controllers (93–95), LN CD8^+^ T cell cytotoxicity *in situ* may be proportional to local HIV replication and antigen expression in spontaneous controllers. We imaged formalin-fixed paraffin embedded (FFPE) LN tissue sections stained for HIV *gagpol* RNA using RNAscope (96, 97) and for CD8, perforin, and granzyme B using immunofluorescence microscopy. HIV RNA was detected at variable levels above background in most LNs, including those from spontaneous controllers without detectable plasma viremia, and was generally higher among untreated controllers than ART-suppressed noncontrollers independent of plasma viremia (fig. S4A**-**C). Consistent with prior reports (20, 38), the majority of HIV replication was confined within follicles of HIV controllers (figs. 6A, S4A), which largely but incompletely excluded CD8^+^ cells (fig. 6A). Intrafollicular CD8^+^ cells often appeared in clusters with elevated perforin and granzyme B expression near HIV RNA^+^ cells (fig. 6A, insets), consistent with antigen-induced proliferation and upregulation of cytotoxic effector molecules among HIV-specific CD8^+^ T cells from spontaneous controllers (fig. 1F). Densities of CD8^+^ cells co-expressing perforin and granzyme B and of cells expressing HIV RNA were quantified across whole LN tissue sections from spontaneous controllers, revealing a positive association between HIV replication and CD8^+^ T cell cytolytic effector molecule expression (fig. 6B).

Because most HIV replication persists within TFH-rich GCs (44) where cytotoxic CD8^+^ T cells have relatively limited access (20, 50–54), we additionally quantified the frequency and spatial localization of CD8^+^ cells expressing cytotoxic effector molecules within spontaneous controller GCs using quantitative image analysis software application. Follicle and GC boundaries were defined by morphology, relative paucity of CD8^+^ cells, and IgD^+^ follicular mantle zone B cells (fig. 6C). Among controllers, we observed a positive correlation between the frequencies of fCD8s co-expressing perforin and granzyme B and cells expressing HIV RNA within GCs (fig. 6D), indicating that fCD8s express cytolytic effector molecules proportional to ongoing intrafollicular HIV replication.

**Fig. 7.**
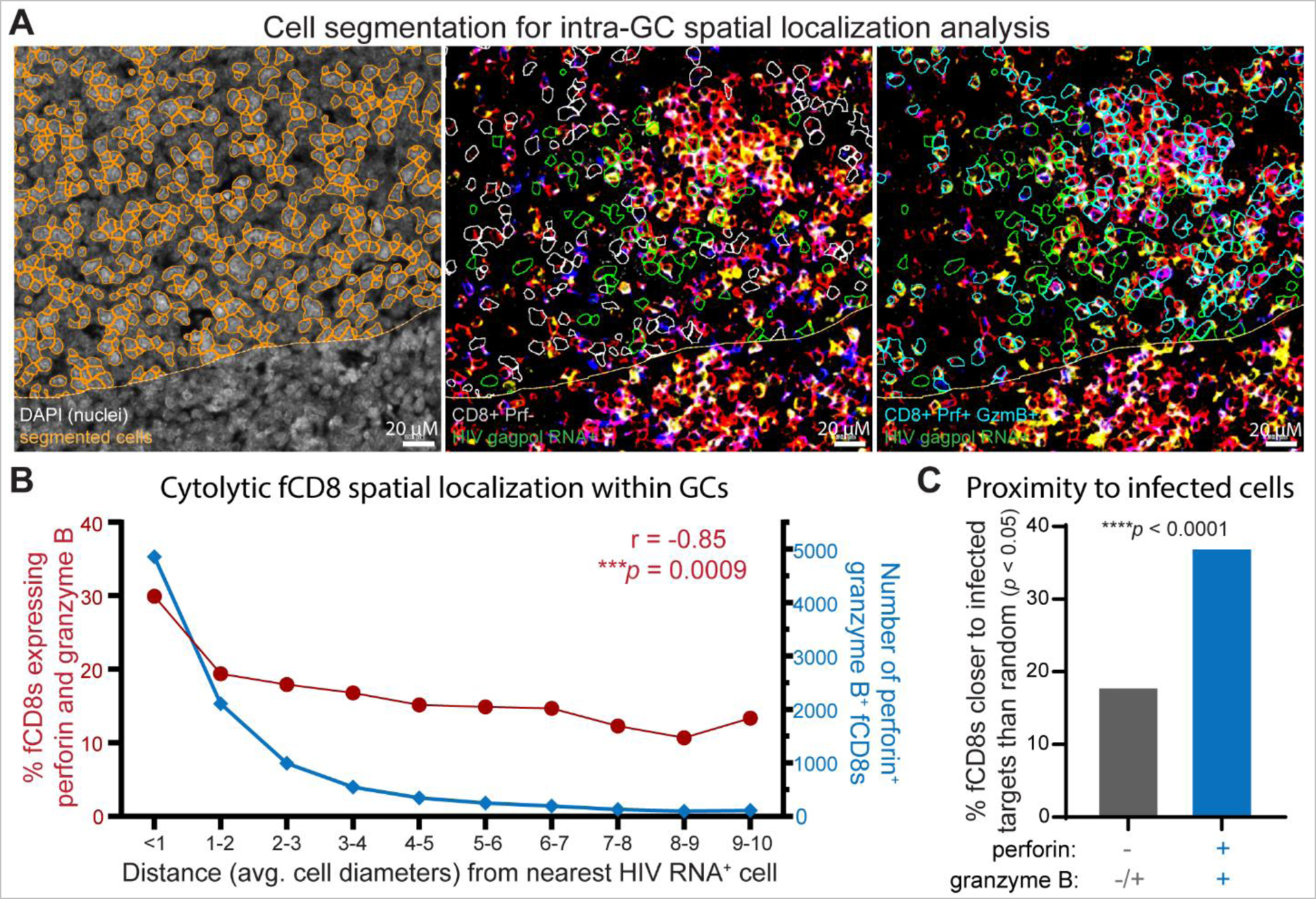
fCD8s express cytolytic effector molecules proximal to HIV-infected cells in GCs. **(A)** Representative IF and RNAscope micrographs depicting automated nuclear segmentation to identify cell boundaries within follicular GC ROI based on DAPI nuclear staining (orange masks, left). Identification of HIV *gagpol* RNA^+^ (green masks), CD8^+^ perforin− (gray masks, center) or CD8^+^ perforin^+^ granzyme B^+^ (cyan masks, right) cells within follicular GC ROI demarcated based on staining as in fig. 6C. **(B)** fCD8s co-expressing perforin and granzyme B within the indicated distance ranges (as average cell diameters corresponding to 8.3 μm increments) from the nearest HIV *gagpol* RNA^+^ cell, expressed as number of cells (right axis, cyan) and percent of total fCD8s within each distance range (left axis, red, Spearman correlation) among 57,606 fCD8s within 52 GCs from 6 controller LNs as in fig. 6D. **(C)** Percent of noncytolytic (perforin–, gray) and cytolytic (perforin^+^ granzyme B^+^, cyan) fCD8s observed significantly (*p* < 0.05) closer to nearest HIV *gagpol* RNA^+^ cell than computationally simulated random effector cell positions. X^2^ test with Yates’ correction (X^2^ = 1931.4, *df* = 1, *n* = 57,606) was used to calculate *p* value for between-group comparison.

To further evaluate spatial relationships between HIV replication and fCD8 cytolytic potential within GCs, we obtained positional information for HIV RNA-expressing cells and for cytolytic (perforin and granzyme B co-expressing) and noncytolytic (perforin-negative) fCD8s using immunofluorescence segmentation (fig. 7A). The proportion of CD8^+^ cells expressing cytotoxic effector molecules was elevated with increasing proximity to HIV RNA^+^ cells (fig. 7B), with the highest expression observed among effector cells within one cell diameter from the nearest HIV RNA^+^ target cell, consistent with antigen-induced upregulation of cytolytic activity among HIV-specific fCD8s in spontaneous controllers. We next compared observed effector-target distances against computationally simulated distances between HIV RNA^+^ target cells and randomly positioned effector CD8^+^ T cells within each GC. Across all GCs analyzed, 37% of cytolytic effectors were observed significantly (*p* < 0.05) closer than random versus only 18% of noncytolytic effectors (fig. 7C), further supporting a role for cytolytic activity in limiting ongoing HIV replication within GCs. Collectively, these data indicate that fCD8s express cytotoxic effector molecules proximal to foci of active HIV replication *in situ* to maintain cytolytic control of intrafollicular viral replication.

## DISCUSSION

This study provides evidence in support of a mechanism of spontaneous immune control of infection by which virus-specific CD8^+^ T cells infiltrate follicular sites of ongoing viral replication in LN, where they proliferate and express cytolytic effector molecules in response to antigen recognition. Our results suggest that inflammatory chemotaxis and antigen-driven proliferation can overcome a relative immune privilege that otherwise limits infiltration and cytotoxicity of CD8^+^ T cells within GCs during homeostasis, highlighting both CXCR5-mediated access and sustained cytolytic effector potential as key features of successful immune control. In contrast, virus-specific CD8^+^ T cells from noncontroller LNs exhibited low CXCR5 expression, poor antigen-specific proliferation and weak cytolytic potential, highlighting important barriers to widespread HIV control or eradication.

CD8^+^ T cell specificity against immutable viral epitopes (98, 99) and a variety of functional attributes including polyfunctionality (100), immediate cytotoxicity (101, 102), proliferative capacity and sustained cytotoxicity (73–79) have been reported as strong correlates of spontaneous HIV control based upon extensive studies of PB (reviewed in (36)). CXCR5-mediated localization of virus-specific CD8^+^ T cells to tissue sites of viral persistence has emerged as an additional correlate of spontaneous HIV control (13–18). However, the precise relationships between circulating and LN-resident fCD8s and the functional attributes associated with long-term control of viremia remain unclear. Reports of immediate cytotoxicity and ex vivo expression of cytolytic effector molecules among virus-specific CD8s in LN have been inconsistent (10–14, 17–19, 53–61, 63–69), perhaps due to differences in disease stage. Short-lived effector cells respond to uncontrolled infection, but ultimately fail to sustain cytolytic control of infection due to defective self-renewal that is not rescued by ART (103). At the other extreme, when HIV infection is controlled to consistently undetectable levels, immediate cytotoxicity is reduced in PB (93–95) and further suppressed in LN (14), spurring investigations into alternative noncytolytic mechanisms of control (104). We provide evidence that in long-term spontaneous controllers, cytolytic effector molecule expression is upregulated proportionally and in close proximity to viral replication in GCs, which occurs even in the absence of detectable plasma viremia. Unlike CD8^+^ T cells from noncontrollers, those from spontaneous controllers exhibited robust capacity to proliferate and upregulate cytolytic effector molecules irrespective of viral burden or location. Together, these results support a tightly regulated mechanism by which fCD8s maintain spontaneous control of infection via antigen-driven proliferation and transient cytotoxicity at sites of ongoing viral replication.

In addition to the functionality of fCD8s, the relative contributions of circulating and noncirculating tissue-resident memory (T_RM_) cells to viral control in LN are also incompletely defined. The extent to which antigen-specific T cells are compartmentalized between LN and PB is variable across reports from different settings. In animal models, transient exposure to antigen via immunization led to clonotypic drift over time between LN and PB in the absence of antigen re-exposure (105), whereas clonotypic composition became more similar over time during chronic viral infection (106). We found complete clonotypic overlap and strikingly high compositional similarity among immunodominant HIV antigen-specific circulating and LN-resident CD8^+^ T cells, suggesting minimal compartmentalization and infiltration of circulating cells. In different settings, other groups observed higher degrees of compartmentalization (21, 69, 107), perhaps due to differences in sampling and/or recent antigen exposure. Nonetheless, we found clear signatures of tissue retention among HIV-specific CD8^+^ T cells in LN, consistent with prior reports in lymphoid tissues (69, 85, 108). These paradoxical findings can be explained by a sentinel function of lymphoid T_RM_ cells to sense antigen and recruit circulating T cells via inflammatory chemotaxis, as has been previously described for nonlymphoid TRM cells (7). Indeed, CCR5- and CXCR3-mediated inflammatory chemotaxis of CD8^+^ T cells to infected LNs has been demonstrated in various infection models (109–113), including HIV (114).

Prior studies demonstrated a relative immune privilege in LN follicles with limited access and functional suppression of CD8^+^ T cells, posing a significant obstacle to HIV cure and remission strategies (9). Multiple reports showed elevated CXCR5^+^ fCD8s in virus controllers that can overcome this barrier to access (13–18), which our results further corroborate and extend. Unlike total CD8^+^ T cells in LN from noncontrollers (115), we demonstrate that cytolytic fCD8s are localized adjacent to foci of HIV replication during spontaneous HIV control. Effector-to-target ratios in LN were correlated with the magnitude of viral suppression in animal models (116) and depletion of CD8^+^ T cells revealed increases in both intrafollicular and extrafollicular HIV replication (19, 20), highlighting their antiviral activity in both regions. Strategies to overcome barriers to follicular surveillance via CXCR5 induction or IL-15 agonism have thus far been unable to successfully reduce viral burden (117, 118). Our results suggest that in addition to follicular access, an additional barrier to cytolytic potential in HIV noncontrollers must also be surmounted, as CD8^+^ T cells from noncontrollers were unable to proliferate and upregulate cytolytic effector molecules in response to antigen re-exposure. CXCR5^+^ CAR T cells have shown promise in reducing follicular viral burden in lymphoid tissue explants (119) and represent one potential strategy to overcoming both barriers. Intriguingly, antigen-specific proliferation in controllers may be intrinsically linked not only to cytolytic effector function (73) but also to CXCR5 upregulation (120), LN homing and infected cell lysis (121). Our data suggest that these potentially interrelated features support long-term immune control of lymphotropic infection by facilitating fCD8 surveillance and sustained cytolytic capacity in response to localized viral recrudescence in GCs.

While our study offers clear insights into HIV-specific CD8^+^ T cells during spontaneous control of viral replication in lymphoid tissue, there were multiple noteworthy limitations. Sample sizes were limited by availability of specimens and interruptions in subject recruitment caused by the COVID-19 pandemic. pHLA tetramer staining *in situ* was not feasible in formalin-fixed tissue, consistent with prior literature (122); however, we were able to study HIV-specific fCD8s using flow cytometry on disaggregated tissue paired with total fCD8 imaging at sites of viral replication. While we were unable to obtain sufficient HIV-specific CD8^+^ T cells from noncontrollers for direct transcriptomic comparisons with controllers, we were able to make such comparisons at single-cell resolution by flow cytometry and functional assays. We were unable to recruit noncontroller participants with untreated chronic HIV infection, but it will be important for future studies to investigate HIV-specific CD8^+^ T cell responses in LN following treatment interruption in individuals who succeed or fail in achieving post-treatment control of viremia. As a proxy, we studied responses to *in vitro* viral antigen re-exposure and noted clear differences in cytolytic potential between controllers and noncontrollers. Likewise, although the data are consistent with augmentation of noncirculating TRM cells by circulating CD8^+^ T cells via inflammatory chemotaxis, dynamic tracking of antigen - specific cells will be required to obtain definitive evidence. Our ability to directly measure cell killing was limited by quantities of available cells and low frequencies of HIV-specific cells; however, results from one durable controller with sufficient LN cells revealed robust cytotoxic elimination upon antigen-specific expansion of LN CD8^+^ T cells (79). Our results highlight the need for additional studies designed to address precise determinants of T cell compartmentalization and immune suppression, and potential relationships between CD8^+^ T cell differentiation, CXCR5 expression and cytolytic potential. We provide evidence that both GC access and sustained cytolytic potential are key determinants of long-term viral control in LN, which will inform efforts to elicit immune control of lymphotropic infections and cancers.

## MATERIALS AND METHODS

### Study design

Contemporaneous PB and excisional inguinal LN biopsy specimens were collected after written informed consent and study approval by the Institutional Review Board of Massachusetts General Hospital (Boston, MA). Viral loads and complete blood count records were obtained from healthcare providers with subject consent. Spontaneous controllers (*n* = 19) maintained plasma viral loads below 2,000 HIV RNA copies/mL (median 27 copies/mL) without ART for a median of 23 years (IQR 12 years) before sample collection. Noncontrollers (*n* = 17) and HIV-negative persons (*n* = 7) were also included in the study. Noncontrollers treated with a variety of standard ART regimens for a median of 12 years (IQR 10 years) maintained undetectable viral loads across longitudinal measurements. Demographic and clinical characteristics of study participants are reported in table 1. *HLA* genotyping was performed by Dr. Mary Carrington (National Cancer Institute, Bethesda, MD). Sample sizes were determined by specimen availability.

### Specimen processing

Inguinal LN biopsy tissue was obtained surgically with informed consent. A portion of tissue was processed into formalin-fixed paraffin-embedded (FFPE) sections for microscopy, and the remainder was mechanically dissociated for cellular assays. Plasma and density gradient isolated PBMCs and cells dissociated from LN were cryopreserved in liquid nitrogen. Frozen cells were thawed at 37°C and recovered in RPMI supplemented with 10% fetal bovine serum (FBS, Sigma) overnight prior to functional and phenotypic assays.

### CD8^+^ T cell response identification

Mononuclear cells were resuspended at 1×10^6^/mL in RPMI supplemented with 10% FBS (R10) and plated 200 μL per well in Immobilon-P 96-well microtiter plates (Millipore) pre-coated with 2 μg/mL anti-IFN-γ (clone DK1, Mabtech). Individual HLA-optimal HIV peptides matched to each subject’s *HLA* genotype (listed in table S1) were added at 1 μM and incubated at 37°C overnight. Negative control wells did not receive peptide and positive control wells were treated with 1 μg/mL anti-CD3 (clone OKT3, Biolegend) and 1 μg/mL anti-CD28 (clone CD28.8, Biolegend) antibodies. ELISPOT assay was performed using manufacturer’s protocol with anti-IFN-γ (clone 1-DK1, Mabtech) capture, biotinylated anti-IFN-γ (clone B6–1, Mabtech) detection, Streptavidin-ALP (Mabtech) and AP Conjugated Substrate (BioRad) followed by disinfection with 0.05% Tween-20 (Thermo Fisher) and analysis using S6 Macro Analyzer (CTL Analyzers). Responses greater than 10 spots per well or 3-fold above negative controls were scored as positive. The largest responses with available pHLA tetramer reagents were selected for downstream investigation of HIV-specific CD8^+^ T cell responses in each individual, listed in table S2.

### Proliferation assay

Mononuclear cells were stained at 37°C for 20 minutes with 0.5 μM CellTrace CFSE (Thermo Fisher) as per manufacturer’s protocol at 1×10^6^ cells/mL. Staining was quenched with FBS (Sigma), cells were washed twice with R10, resuspended at 1×10^6^/mL and plated 200 μL per well in 96-well round-bottom polystyrene plates (Corning). Individual HIV peptides corresponding to IFN-γ ELISPOT responses for each participant were added at 1 μM and incubated at 37°C for 6 days before flow cytometric assessment. Negative control wells did not receive peptide and positive control wells received 1 μg/mL anti-CD3 (clone OKT3, Biolegend) and anti-CD28 (clone CD28.8, Biolegend) antibodies. On day 6, cells were stained for viability using Live/Dead Violet (Thermo Fisher), AlexaFluor700-anti-CD3 (clone SK7, Biolegend), BUV395-anti-CD8 (clone RPA-T8, BD Biosciences), and APC-pHLA tetramer matching the peptide used for stimulation, then analyzed by flow cytometry. Representative gating and controls are shown in fig. S5B.

### Cytolytic degranulation assay

*Ex vivo* (d0) and peptide-expanded (d6) CD8^+^ T cells were isolated from PBMCs and LNMCs using EasySep Human CD8^+^ T Cell Isolation Kit (StemCell Technologies) and stimulated with 4 nM APC-pHLA tetramer at 37°C for 4 hours in R10 with BV711-anti-CD107A (clone H4A3, Biolegend) to measure cytolytic degranulation. Cells were stained with BUV395-anti-CD8 (clone RPA-T8, BD Biosciences), Live/Dead Blue (Thermo Fisher), fixed and permeabilized with Cytofix/Cytoperm (BD Biosciences), and stained for intracellular PE-anti-perforin (clone B-D48, Biolegend) and PE-CF594-anti-granzyme B (clone GB11, BD Biosciences). FMO controls were used to establish gating. Experimental negative controls were pre-treated with 50 nM dasatinib 30 minutes before tetramer stimulation to prevent activation, and experimental positive controls were treated with PMA and ionomycin (eBioscience Cell Stimulation Cocktail, Thermo Fisher). Samples were analyzed by flow cytometry. Representative gating and controls are shown in fig. S5A and C. Samples with fewer than 40 live HIV-specific pHLA-tetramer^+^ CD8^+^ T cells at d0 in both PB and LN were excluded from downstream analyses.

### Phenotypic cytometry

Peptide-HLA monomers were obtained from ImmunAware (Copenhagen, Denmark). Tetramers were produced by multimerization with APC-conjugated streptavidin (Biolegend) as per manufacturer’s protocol and stored at 4°C for a maximum of 4 weeks prior to use. Staining was performed using 4 nM individual APC-conjugated pHLA tetramers at 4°C for 30 minutes after 30-minute pre-treatment with 50 nM dasatinib to prevent *in vitro* cell activation, degranulation and activation-induced cell death for phenotypic analysis. Cells were then stained with Live/Dead Blue viability dye (Thermo Fisher), BUV395-conjugated anti-CD8 (clone RPA-T8, BD Biosciences), BV421-anti-CD45RA (clone HI100, Biolegend), BV650-anti-CD62L (clone DREG-56, Biolegend), BV711-anti-CCR7 (clone G043H7, Biolegend), PerCP/Cy5.5-anti-CD56 (clone 5.1H11, Biolegend) and PE/Cy7-anti-CXCR5 (clone J252D4, Biolegend) for 30 minutes, after which cells were fixed and permeabilized (Cytofix/Cytoperm, BD Biosciences) and stained for intracellular markers using PE-anti-perforin (clone B-D48, Biolegend) and PE/CF594-anti-granzyme B (clone GB11, BD Biosciences) for 30 minutes. In some experiments, cells were instead stained with Live/Dead Blue, BUV395-anti-CD8, BV421-anti-CD45RA, BV650-anti-CD62L, AlexaFluor 488-anti-CXCR5 (clone RF8B2, BD Biosciences), PE/Cy7-anti-PD-1 (clone A17188B, Biolegend), AlexaFluor 700-anti-CD69 (clone FN50, Biolegend), PE-anti-CD103 (clone B-Ly7, Thermo Fisher) and PE/eFluor 610-anti-CX3CR1 (clone 2A9–1, Thermo Fisher) or with PE-anti-CCR5 (clone T21/8, Thermo Fisher) and PE/Dazzle 594-anti-CXCR3 (clone G025H7, Biolegend) before fixation. Cells were analyzed by flow cytometry. Gating thresholds were established using appropriate negative controls, including fluorescence-minus-one and biological negative controls, as shown in fig. S5. Gates were applied consistently across all intra- and inter-sample comparisons. Samples with viability below 50% or fewer than 50 live HIV-specific pHLA-tetramer^+^ CD8^+^ T cells were excluded from downstream analyses.

### Flow cytometry and FACS

Flow cytometry and cell sorting were performed at the Ragon Institute flow cytometry and imaging core using BD Fortessa and LSR-II cytometers, FACSAria sorters and FACSDiva software (Becton Dickinson). Flow cytometric analyses were performed using FlowJo v10.8 (TreeStar).

### TCR and RNA-sequencing

8 study participants, including 7 spontaneous controllers and 1 ART-suppressed noncontroller were selected for analysis on the basis of specimen and pHLA^+^ tetramer availability. Replicate populations of 500 – 2,500 HIV-specific CD8^+^ T cells were sorted by FACS on the basis of staining with individual pHLA tetramers into RLT Plus lysis buffer then frozen at −80 C. The following were performed as previously described (79): RNA isolation using AllPrep (Qiagen), whole transcriptome amplification by SmartSeq2, tagmentation using Nextera XT (Illumina), sequencing on NextSeq 550 (Illumina) using 75 base paired end reads, quality control using FastQC, alignment to hg38 reference human transcriptome and transcript quantitation using RSEM/bowtie1, alignment and filtering of T cell receptor beta (*TRB*) complementarity determining region 3 (CDR3) sequences using MiXCR, exclusion of genes with fewer than 5 reads in fewer than 10% of samples, multi-factor linear modeling to isolate biological effects of interest (e.g., anatomical source) from potentially confounding sources of variation (e.g., participant heterogeneity, sampling error) via forward model selection using AIC and filtering for co-linear factors for differential expression analysis using DESeq2, gene set enrichment analyses using CERNO ranked by FDR-adjusted *q* values (signed by direction of fold change) with GO:BP, GO:CC, GO:MF, Reactome, Kegg and Hallmark gene sets from MSigDB, and organization of differentially expressed gene sets into directional nearest-neighbor networks using gene set network analysis (GSNA). TCR clonotypes below 2% of total *TRB* reads were grouped as “other” due to lack of concordance across replicates below this threshold and the possibility of nonspecific tetramer binding at low frequencies representing potential single cells. Enriched transcription factor (TF) regulation signatures in PB and LN were identified by CERNO pathways analysis on TF-associated activated and repressed target gene sets derived from the dorothea human non-cancer A, B and C confidence database (87). Differentially enriched TF gene sets were organized into a hierarchical network based on member target genes using GSNA (79). Single-sample gene set enrichment analyses (ssGSEA) were performed for five gene sets associated with interferon responsiveness (genes with prefixes *IFN*, *ISG* and *IFI*), LN follicular homing [*CXCR5, SLAMF6, SELL, TCF7, ID3, CD200, ICOS, IL7R, BCL6* (12)], virus-specific CD8^+^ T cell activation [*CRTAM, IFNG, XCL2, TNFRSF9, XCL1, EGR2, MIR155HG, IL2RA, TNFRSF18, CTNNA1, CD82, TAGAP, HSP90AB1, ACTG1, HSPA8, GZMB, GNG4, NR4A1, ACTB, PKM* (84)], CD8^+^ T cell tissue retention [*CA10, IL17A, CXCL13, SCUBE1, GSG2, ITGA1, CXCR6, ATP8B4, CSF1, ITGAE, CPNE7, IL10, SPRY1, MCAM, RGS1, KCNQ3, DAB2IP, TRPM2, KCNK5, IL23R, PELO, COL5A1, IRF4, FSD1, IL17RE, ADAM12, CRTAM, ARHGAP18, CCR1, AMICA1, ICOS, TMIGD2, TP53INP1, BMF, CD9, RIMS3, DUSP6, CCR6, GZMB, ZNF683* (85)] and tissue-emigrant CD8^+^ T cell lymphatic recirculation [*LC17A2, SORCS3, NRG1, COQ3, REG4, SCUBE1, PJA2, FCRLA, LINC02225, CERS5, APOF, MAX, URAHP, PCDH11Y, CEP68, MAT2A, NMU, C8G, CARS2* (86)] in our data set.

### Immunofluorescence and RNAscope imaging

FFPE inguinal LN tissue sections were stained with DAPI (nuclear marker), mouse IgG1 anti-perforin (clone 5B10, Leica), mouse IgG2a anti-granzyme B (clone GB7, BioRad), polyclonal rabbit anti-CD8 (Abcam) and/or polyclonal goat anti-IgD (Southern Biotech) primary antibodies followed by AlexaFluor550-anti-mouse IgG1, AlexaFluor488-anti-mouse IgG2a (Thermo Fisher), AlexaFluor750-anti-rabbit IgG and/or AlexaFluor488-anti-goat IgG (R&D Systems) secondary antibodies. HIV RNA was detected using RNAscope Multiplex Fluorescent Reagent Kit v2 using a HIV *gagpol* probe and Cy5.5-conjugated amplifiers (immunofluorescence images) or ALP-conjugated amplifiers with Fast-Red substrate (chromogenic images) as per manufacturer’s protocol (Advanced Cell Diagnostics). Fluorescence specificity and background staining were established using manufacturer’s negative and positive control staining probes (Advanced Cell Diagnostics) and by staining of an HIV-uninfected LN. Stained sections were mounted on SuperFrost microscopy slides (Thermo Fisher) and imaged using the TissueFAXS automated slide scanning system (TissueFAXS USA) consisting of a Zeiss Axio Imager Z2 upright microscope, a Lumencor Spectra 3 light engine (Lumencor, Inc.), combined with a Zeiss 20X 0.5NA Plan-Neofluar objective and a Maerzhauser automated microscope stage.

Images were processed using TissueFAXS Viewer (TissueGnostics USA) and analyzed using StrataQuest analysis software (TissueGnostics USA) to segment and quantify DAPI^+^ cells expressing HIV *gagpol* RNA and DAPI^+^ cells co-expressing CD8, perforin and granzyme B across entire immunostained tissue sections. To evaluate intrafollicular events, a custom StrataQuest application was developed by TissueGnostics. Follicular GC regions of interest (ROIs) were drawn manually based upon morphology, relative CD8 exclusion and IgD staining of mantle-zone B cells in adjacent tissue sections, as illustrated in fig. 6C. Nuclei were computationally segmented based on DAPI staining and refinement of cellular masks using CD8, perforin and granzyme B staining as shown in fig. 7A. The threshold for HIV *gagpol* RNA detection above background was determined using RNAscope staining of HIV-uninfected LNs as shown in fig. S3B, and thresholds for CD8, perforin and granzyme B detection were set based on isotype controls. To evaluate distances of cytolytic and noncytolytic fCD8s from the nearest HIV *gagpol* RNA positive cell, x-y coordinates of HIV *gagpol* RNA positive cells, perforin^+^ granzyme B^+^ CD8^+^ cells and perforin^−^ CD8^+^ cells were extracted from image analysis reports. To compare observed effector-to-nearest-target distances against a theoretical uniform distribution, we simulated random placement of CD8^+^ effector cells 100,000 times within each ROI while retaining HIV RNA^+^ target cell positions and overall GC geometry. Observed distances were compared to simulated distances for each CD8^+^ effector cell to determine the probability that randomly positioned effector CD8^+^ cells would be located at the same distance or closer to the nearest HIV RNA^+^ target cell than observed.

### Statistical analyses

Statistical tests were performed using GraphPad Prism v9.0.2 and R. Parametric tests were used for data with normal distributions and nonparametric tests were used for data with non-normal distributions. For data with seven or more replicates, normality was determined using Shapiro-Wilk tests. For data with fewer than seven replicates, normality was estimated by skewness. Where indicated, *p* values were false discovery rate adjusted (*q*) using the Benjamini-Hochberg method. *p* or *q* values less than 0.05 were considered statistically significant. Figures were prepared using GraphPad Prism v9.0.2 and Adobe Illustrator CC v22.0.1. Tables were prepared using Microsoft Word and Excel.

## Supplementary Material

Supplementary Materials SUPPLEMENTARY MATERIALS
Fig. S1. Identification of HIV-specific CD8^+^ T cells.
Fig. S2. Gene set network enrichment in LN and PB HIV-specific CD8^+^ T cells.
Fig. S3. Flow cytometric phenotyping of circulating and LN-derived CD8^+^ T cells.
Fig. S4. HIV RNA *in situ* hybridization imaging and quantitation.
Fig. S5. Flow cytometric gating schema and staining controls.
Table S2. Immunodominant HIV antigen-specific responses.

Table S1 Table S1. HLA-optimal HIV peptides.

Table S3 Table S3. Differentially expressed genes.

Table S4 Table S4. Differentially expressed pathways and transcription factor signatures.

Table S5 Table S5. Tabular data for statistical tests.

## Figures and Tables

**Table 1:** Participant demographics and clinical parameters.

		All	Controllers	ART	HIV-
***n***		43	19	17	7
**Age** (median, *IQR****)***		53 (13)	57 (16)	53 (9)	47 (18)
**Sex *(****n*, *%****)***	*Female*	7 ***(****16.3%****)***	2 ***(****10.5%****)***	4 (23.5%)	1 (14.3%)
	*Male*	35 ***(****83.7%****)***	17 ***(****89.5%****)***	13 (76.5%)	6 (85.7%)
**Ethnicity *(****n*, *%****)***	*African American*	14 ***(****32.6%****)***	7 ***(****36.8%****)***	7 (41.2%)	0 (0%)
	*Caucasian*	27 ***(****62.8%****)***	12 ***(****63.2****%)***	8 ***(****47.1%)*	7 (100%)
	*Hispanic/Latino*	1 (2.3%)	0 (0%)	1 (5.9%)	0 (0%)
	*Native American*	1 (2.3%)	0 (0%)	1 (5.9%)	0 (0%)
**CD4+ T Cells** (cells/μL blood; median, *IQR****)***		753 ***(****539****)***	912 ***(****478****)***	554 (503)	N/A
**Plasma Viral Load** (HIV RNA copies/mL; median, *IQR****)***		<20 (10)	27 (85)	<20 (0)	N/A
**Undetectable Plasma Viral Load *(*** *n, %* ***)***		24 (55.8%)	8 ***(****42.1%****)***	16 (94.1%)	N/A
**Peak Pre-ART Viral Load** ***(****n, %,* median HIV RNA copies/mL)		N/A	N/A	n=8 ***(****47%****)*** 3.0×10^5^	N/A
**Protective *HLA-B* Alleles** (n, %) [B*14, B*27, B*52, B*57, B*58, B*81]		18 ***(****50%****)***	14 ***(****73.7%****)***	4 (21.1%)	N/A
**Duration of Control** (years; median, *IQR*; *by ART)		N/A	23 (12)	12* (10)	N/A

## DATA AND MATERIALS AVAILABILITY

The accession number for RNA-sequencing data generated in this study is GEO:GSE196549. The remaining data supporting the findings of this study are available in the article, its supplementary information files, and/or from the corresponding author by request.
