# Supplementary material for: Cytolytic CD8+ T cells infiltrate germinal centers to limit ongoing HIV replication in spontaneous controller lymph nodes
Source: Sci Immunol. Author manuscript; Available in PMC 2023 May 31. (PMC10231436; doi:10.1126/sciimmunol.ade5872)
Supplement: Supplementary Materials — Fig. S1. Identification of HIV-specific CD8+ T cells. Fig. S2. Gene set network enrichment in LN and PB HIV-specific CD8+ T cells. Fig. S3. Flow cytometric phenotyping of circulating and LN-derived CD8+ T cells. Fig. S4. HIV RNA in situ hybridization imaging and quantitation. Fig. S5. Flow cytometric gating schema and staining controls. Table S2. Immunodominant HIV antigen-specific responses. [file NIHMS1903002-supplement-Supplementary_Materials.pdf]

Science Immunology
AAAS

Supplementary Materials for

Cytolytic $CD8^+$ T cells infiltrate germinal centers to limit ongoing HIV replication in spontaneous controller lymph nodes

David R. Collins *et al.*

Corresponding author: Bruce D. Walker, bwalker@mgh.harvard.edu

Sci. Immunol. **8**, eade5872 (2023)
DOI: 10.1126/sciimmunol.ade5872

The PDF file includes:

Figs. S1 to S5
Table S2
Legends for tables S1, S3 to S5

Other Supplementary Material for this manuscript includes the following:

Tables S1, S3 to S5
MDAR Reproducibility Checklist

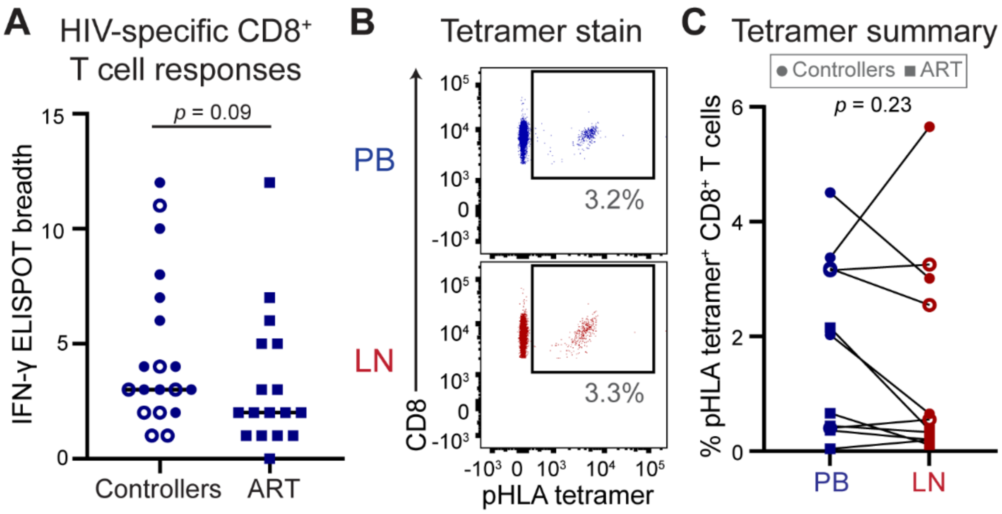

Fig. S1. Identification of HIV-specific CD8⁺ T cells. (A) Breadth of CD8⁺ T cell responses detected by IFN-γ ELISPOT of PBMCs from spontaneous controllers (*n* = 19, circles) and ART-suppressed noncontrollers (*n* = 17, squares). Lines represent medians. Mann-Whitney U-test was used to calculate *p* value. **(B)** Representative staining of CD8⁺ T cells from paired PB and LN with pHLA tetramers corresponding to HIVspecific responses identified in A. **(C)** Frequencies of pHLA tetramer⁺ among CD8⁺ T cells in paired PB and LN specimens (*n* = 14). Wilcoxon matched-pairs signed rank test was used to calculate *p* value. Gray box contains symbol key: circles represent controllers, squares ART; open or closed circles denote controllers with undetectable or detectable plasma HIV RNA, respectively.

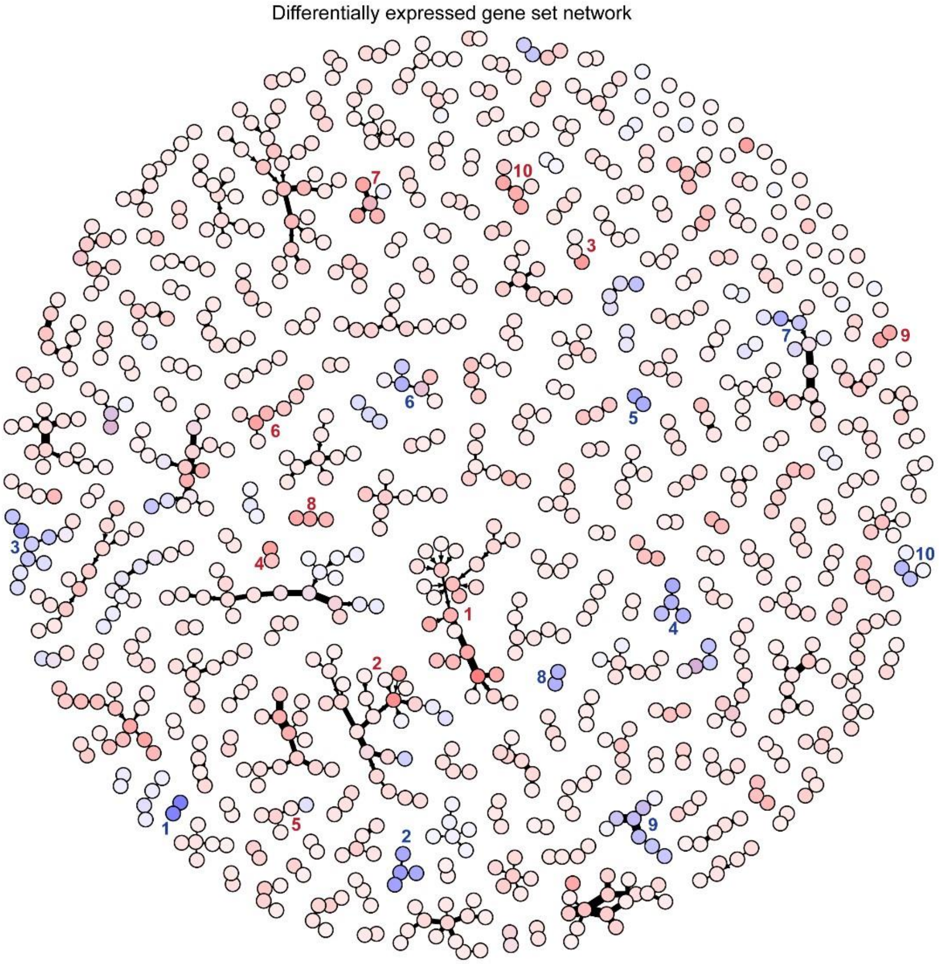

Fig. S2. Gene set network enrichment in LN and PB HIV-specific CD8+ T cells. Diagram representing nearest-neighbor network of significantly differentially expressed gene sets among HIV-specific CD8+ T cells isolated from PB or LN, as generated via gene set network analysis. Top ten differentially expressed gene set subnets are labeled for PB (blue) and LN (red), corresponding to fig. 3C and table S4.

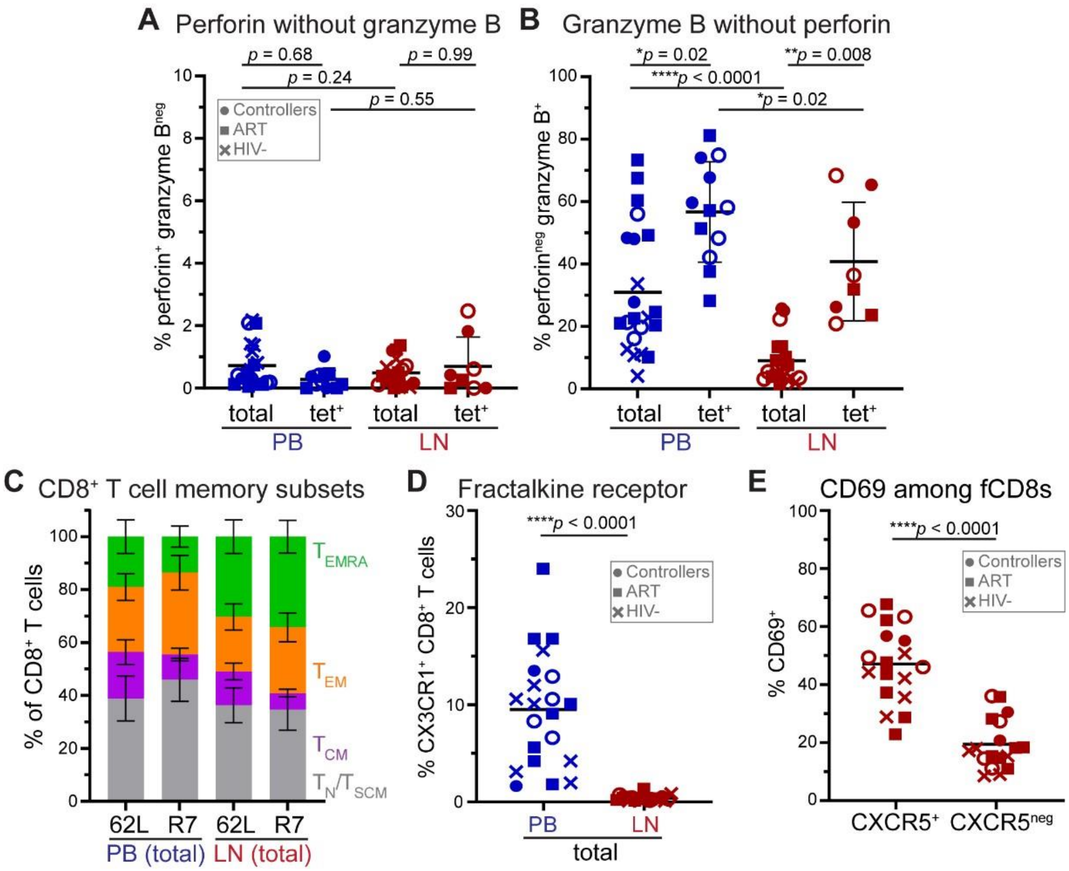

Fig. S3. Flow cytometric phenotyping of circulating and LN-derived CD8⁺ T cells. (A-B) Frequencies of perforin⁺ granzyme B⁻ (A) and perforin⁻ granzyme B⁺ (B) among total and HIV pHLA tetramer⁺ (tet⁺) CD8⁺ T cells in paired PB and LN specimens (n = 21). Wilcoxon matchedpairs signed rank test was used to calculate p values. **(C)** Comparison of memory subset composition defined by CD45RA and CD62L (62L) versus CCR7 (R7) among total (n = 22) and CD8⁺ T cells in PB and LN. Stacked bars represent mean, error bars represent 95% confidence intervals. **(D)** Frequencies of CX3CR1⁺ among CD8⁺ T cells in paired PB and LN specimens (n = 21). Wilcoxon matched-pairs signed rank test was used to calculate p value. **(E)** Frequencies of CD69⁺ among CXCR5⁺ fCD8s vs CXCR5⁻ CD8⁺ T cells in LN specimens (n = 18). Wilcoxon matched-pairs signed rank test was used to calculate p value. Gray boxes contain symbol keys: circles represent controllers, squares ART, crosses HIV⁻; open or closed circles denote controllers with undetectable or detectable plasma HIV RNA, respectively.

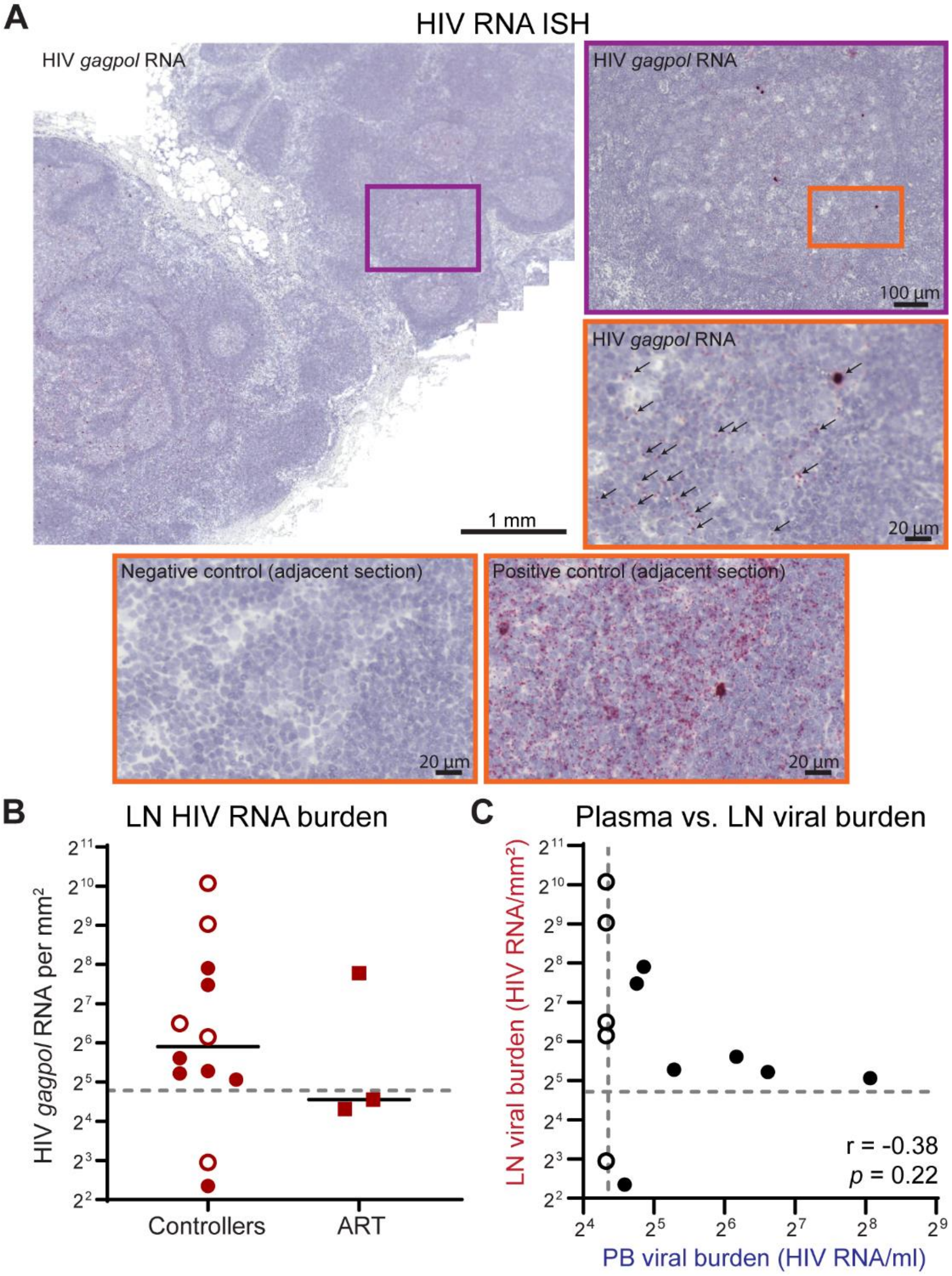

Fig. S4. HIV RNA *in situ* hybridization imaging and quantitation. (A) Representative chromogenic RNAscope ISH of HIV *gagpol* RNA (upper panels), negative or positive control RNAscope probes (lower panels) on adjacent controller LN sections. Arrows highlight HIV *gagpol* RNA staining. **(B)** Summary quantitations of HIV RNA in LNs from controllers, ART-suppressed noncontrollers. Dashed line represents detection limit established by staining HIV-negative LN for HIV *gagpol* RNA**. (C)** No significant Spearman correlation between plasma and LN viral burdens in spontaneous controllers. Open or closed circles denote controllers with undetectable or detectable plasma HIV RNA, respectively. Dashed lines represent limits of detection.

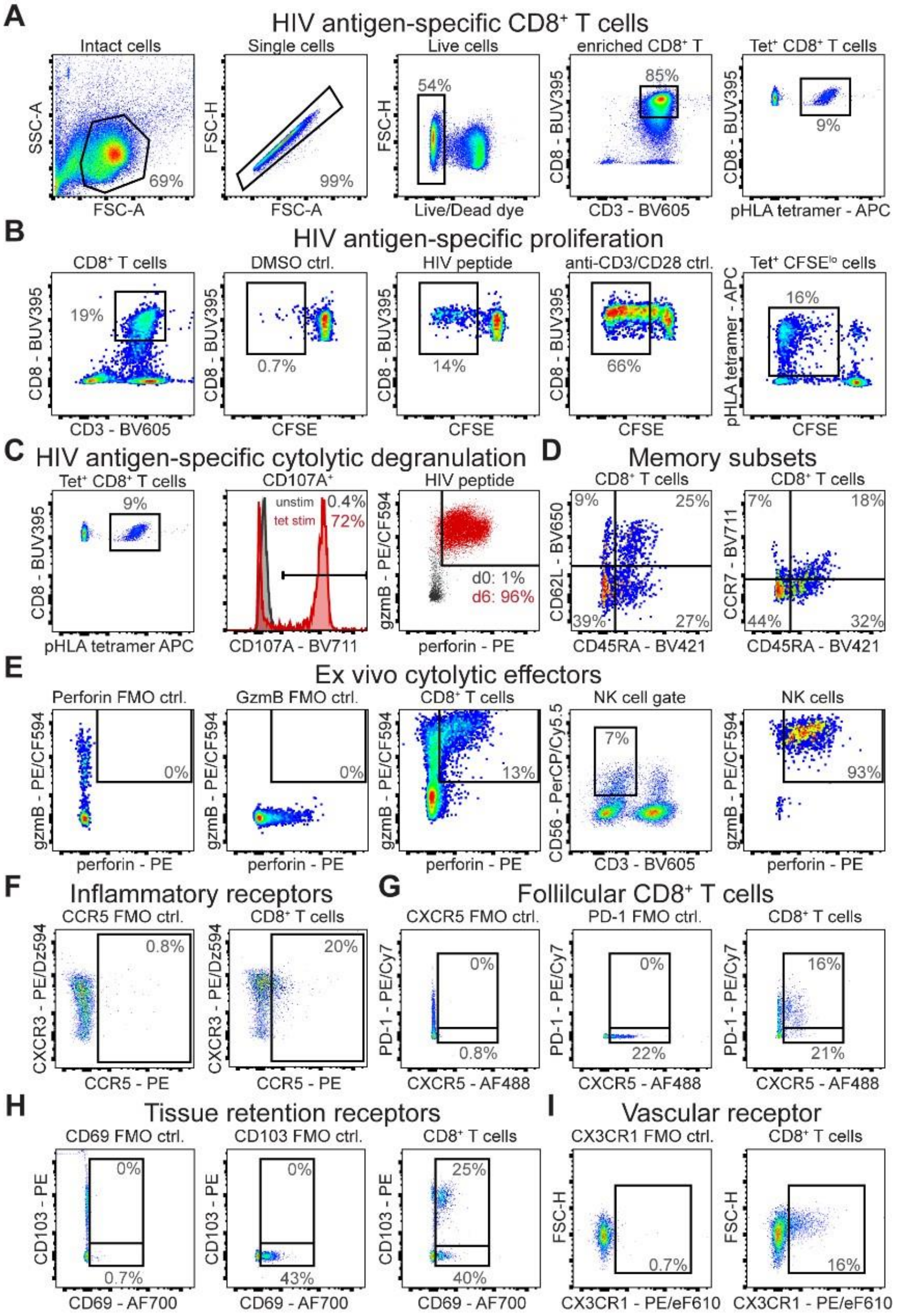

Fig. S5. Flow cytometric gating schema and staining controls. (A) Gating of HIV pHLA tetramer$^+$ CD8$^+$ T cells for downstream analyses. **(B)** Gating (left panel) and representative pseudocolor scatter dot plots for negative (DMSO) and positive (anti-CD3/CD28) controls and immunodominant HIV-specific peptide pulsed CD8$^+$ T cells in CFSE proliferation assays and HIV pHLA tetramer staining on day 6 (right panel) related to fig. 1. **(C)** Gating and representative results of HIV antigen-specific cytolytic degranulation including CD107A upregulation (middle panel) in negative control (unstim) and 4-hour pHLA tetramer stimulated (tet stim) HIV pHLA tetramer$^+$ CD8$^+$ T cells and perforin and granzyme B co-expression (right panel) within CD107A$^+$ tet$^+$ CD8$^+$ T cells upon 4-hour tetramer stimulation *ex vivo* (d0) or after 6-day stimulation with immunodominant HLA-optimal HIV peptide (d6) related to fig. 1. **(D)** Representative gating scheme for CD8$^+$ T cell memory subset classification using CD45RA and CD62L (left) or CCR7 (right) related to figs. 4 and S3. **(E)** Fluorescence-minus-one (FMO) staining controls for *ex vivo* perforin (left panel) and granzyme B (gzmB, second panel) in CD8$^+$ T cells (co-stained, third panel); gating scheme for NK cells (fourth panel) and representative perforin and gzmB expression by NK cells as a positive staining control (right panel) related to figs. 4, 5 and S3. **(F)** CCR5 FMO staining control and representative CD8$^+$ T cell staining for CCR5 and CXCR3 related to fig. 5. **(G)** CXCR5 and PD-1 FMO staining controls and representative staining of CD8$^+$ T cells related to figs. 5 and S3. **(H)** CD69 and CD103 FMO staining controls and representative staining of CD8$^+$ T cells related to figs. 5 and S3. **(I)** CX3CR1 FMO staining control and representative staining of CD8$^+$ T cells related to fig. S3.

Controllers		Noncontrollers	
○ C1	B*57 Gag TW10	NC1	A*02 Pol IV9
○ C2	B*58 Gag TW10	NC2	B*51 Pol LI9
● C3	B*53 Nef YY9	NC3	B*18 Pol NY10
○ C4	B*57 Gag KF11	NC4	C*08 Gag TL9
○ C5	B*57 Gag TW10	NC5	B*35 Pol TY9
● C6	B*57 Gag TW10	NC6	B*57 Gag TW10
○ C7	B*57 Gag KF11	NC7	None Detected
○ C8	B*08 Gag EI8	NC8	A*03 Gag KK9
● C9	B*57 Gag IW9	NC9	B*44 Gag AW11
○ C10	B*57 Gag KF11	NC10	B*08 Gag EI8
○ C11	A*24 Gag KW9	NC11	B*14 Gag DA9
● C12	B*53 Gag QW9	NC12	B*52 Gag RI8
● C13	B*81 Gag TL9	NC13	C*04 Env SF9
● C14	B*57 Gag KF11	NC14	A*03 Env TK10
● C15	B*57 Gag KF11	NC15	B*13 Nef RV9
● C16	B*42 Gag TL9	NC16	B*15 Pol VI10
● C17	B*57 Gag KF11	NC17	B*40 Pol IL8
● C18	B*57 Gag KF11		
● C19	A*26 Gag EL9		

Table S2. Immunodominant HIV antigen-specific responses. List of immunodominant HIV antigen-specific $CD8^+$ T cell responses determined by IFN-γ ELISPOT screening of PBMCs from each participant. For controllers, open and closed circles represent undetectable and detectable plasma viremia, respectively.

Additional Supplementary Files:

Table S1. HLA-optimal HIV peptides. List of HLA-optimal HIV peptides screened for each class I HLA allele and their corresponding HIV protein, selected from Los Alamos National Laboratory database. (See Excel spreadsheet.)

Table S3. Differentially expressed genes. Full list of differentially expressed genes from RNA-seq analysis of HIV-specific $CD8^+$ T cells from LN versus PB, including fold changes and statistics. (See Excel spreadsheet.)

Table S4. Differentially expressed pathways and transcription factor signatures. Subnets of significantly enriched gene sets and transcription factor signatures from MSigDB using CERNO to compare differentially expressed genes upregulated by HIV-specific $CD8^+$ T cells in LN or PB, ranked by directional significance. (See Excel spreadsheet.)

Table S5. Tabular data for statistical tests. Tabular data for all statistical tests with sample sizes less than 25. (See Excel spreadsheet.)
